# Selection following Gene Duplication Shapes Recent Genome Evolution in the Pea Aphid *Acyrthosiphon pisum*

**DOI:** 10.1093/molbev/msaa110

**Published:** 2020-05-02

**Authors:** Rosa Fernández, Marina Marcet-Houben, Fabrice Legeai, Gautier Richard, Stéphanie Robin, Valentin Wucher, Cinta Pegueroles, Toni Gabaldón, Denis Tagu

**Affiliations:** m1 Centre for Genomic Regulation (CRG), The Barcelona Institute for Science and Technology, Barcelona, Spain; m2 Institute for Research in Biomedicine (IRB Barcelona), Barcelona, Spain; m3 IGEPP, INRAE, Agrocampus Ouest, Université de Rennes 1, Le Rheu, France; m4 INRIA, IRISA, Genscale, Campus Beaulieu, Rennes, France; m5 Max Planck Institute of Immunobiology and Epigenetics, Freiburg im Breisgau, Germany; m6 INRIA, IRISA, GenOuest Core Facility, Campus Beaulieu, Rennes, France; m7 Universitat Pompeu Fabra, Barcelona, Spain; m8 Institució Catalana de Recerca i Estudis Avançats (ICREA), Barcelona, Spain; m9 Animal Biodiversity and Evolution, Institute of Evolutionary Biology (CSIC-UPF), Barcelona, Spain; m10 Department of Life Sciences, Barcelona Supercomputing Center, Barcelona, Spain

**Keywords:** gene duplicates, FAIRE-Seq, insect, neofunctionalization, phylogenomics, positive selection

## Abstract

Ecology of insects is as wide as their diversity, which reflects their high capacity of adaptation in most of the environments of our planet. Aphids, with over 4,000 species, have developed a series of adaptations including a high phenotypic plasticity and the ability to feed on the phloem sap of plants, which is enriched in sugars derived from photosynthesis. Recent analyses of aphid genomes have indicated a high level of shared ancestral gene duplications that might represent a basis for genetic innovation and broad adaptations. In addition, there are a large number of recent, species-specific gene duplications whose role in adaptation remains poorly understood. Here, we tested whether duplicates specific to the pea aphid *Acyrthosiphon pisum* are related to genomic innovation by combining comparative genomics, transcriptomics, and chromatin accessibility analyses. Consistent with large levels of neofunctionalization, we found that most of the recent pairs of gene duplicates evolved asymmetrically, showing divergent patterns of positive selection and gene expression. Genes under selection involved a plethora of biological functions, suggesting that neofunctionalization and tissue specificity, among other evolutionary mechanisms, have orchestrated the evolution of recent paralogs in the pea aphid and may have facilitated host–symbiont cooperation. Our comprehensive phylogenomics analysis allowed us to tackle the history of duplicated genes to pave the road toward understanding the role of gene duplication in ecological adaptation.

## Introduction

Aphids are insect pests belonging to the order Hemiptera, which diverged some 280–250 Ma. They feed exclusively on plant phloem sap, a trait that involves specific adaptations such as an obligatory symbiosis with bacteria of the genus *Buchnera*, which supplies aphids with essential amino acids that are missing in the phloem sap. In addition, to adapt to stressful environments such as cold, predation, and parasitism (Vellichirammal et al. 2016), aphids have developed several plastic phenotypic traits, involving winged and apterous morphs, or sexual oviparous and parthenogenetic viviparous female morphs. Although several studies have addressed the genetic mechanisms of these adaptations at the molecular level, the evolutionary forces underlying these genomic changes are still poorly understood. Today, several aphid genomes are publically available, and all show a high level of gene duplication and expansions (International Aphid Genomics Consortium 2010; Mathers et al. 2017; Li et al. 2019). Some of these duplications are shared between aphid species, but most of them are lineage specific (International Aphid Genomics Consortium 2010). The modes of evolution of gene duplicates occurring in these species are not yet fully determined. Whether or not duplicated or expanded gene families are in relation with the above-mentioned or other functional innovations enabling adaptive evolution in aphids is still largely unknown (Huerta-Cepas, Dopazo, et al. 2011; Huerta-Cepas, Marcet-Houben, et al. 2010; Simon et al. 2011).

There are at least four different outcomes for gene duplicates (reviewed by Capella-Gutierrez et al. [2009] and Innan and Kondrashov [2010]). First, although one duplicate keeps the original function, the other acquires a new function (neofunctionalization). Second, each of the two duplicated genes keeps part of the functions of the ancestral gene, so that they jointly cover the original functions (subfunctionalization). Third, when the increase in gene dosage is beneficial, the two copies are maintained in the absence of functional divergence. And fourth, the most common output of gene duplication is the inactivation by accumulation of mutations of one of the duplicated genes (pseudogenization). Several evolutionary forces can drive these different outcomes, for instance relaxed selection for subfunctionalization, purifying selection for neofunctionalization, or deleterious mutations for pseudogenization (Lynch and Conery 2000; Han et al. 2009; Innan and Kondrashov 2010). These different scenarios can be addressed by scrutinizing patterns of variation of gene families including lineage-specific duplications (Han et al. 2009; Innan and Kondrashov 2010; Pegueroles et al. 2013; Pich I Roselló and Kondrashov 2014).

More recently, sub- or neo-functionalization have started to be assessed by epigenetic regulation. Acquiring and losing functions can occur, among other means, by modification of chromatin states, which drive the transcriptional activities of genes. The so-called “open chromatin,” in which accessible DNA allows for active transcription, can be opposed to the so-called “closed” chromatin, which is compact and transcriptionally repressed. Little is known about the role of chromatin in determining the fate of duplicated genes, but it is intuitive to think that two duplicated gene copies could have spatially or temporally different chromatin states, thus resulting in different transcription patterns. For instance, Keller and Yi (2014) showed that the DNA methylation of gene promoters of both copies of young duplicates in humans is higher than that of old duplicates. This observation stands for different tested tissues, indicating that this trait is not related to tissue-specificity regulation, as DNA methylation is known to regulate transcription. Thus, it could be hypothesized that chromatin state influences the expression of duplicated copies—and thus consequently their evolution—possibly as a protection against possible misregulations by dosage compensation (Chang and Liao 2012), before mutations occur and genetic selection operates. It is worth noting that divergent epigenetic environments may result in subfunctionalization (e.g., through changed expression patterns), but the epigenetic differences themselves do not result from functional differences between the copies.

Here, we test the hypothesis that gene duplication—particularly recent duplicates—in the pea aphid *Acyrthosiphon pisum* is a source of innovation fueled by selection. For this, we anchor our study on a phylogenomic approach exploring for the first time ten hemipteran genomes, including six aphid species. We show that 1) a large proportion of gene duplications are under positive selection in *A. pisum* and affect a large number of biological functions (most notably oo- and morpho-genesis and host–symbiont cooperation), 2) asymmetrical rates of young paralogs coupled to positive selection suggest that neofunctionalization is a main force reshaping the pea aphid genome, 3) a third of young duplicates show divergent tissue expression patterns, consistent in some cases with subfunctionalization by tissue specialization and others with neofunctionalization through gain of gene expression, and 4) chromatin accessibility of the transcription start site (TSS) can change between genes in duplicated gene pairs, although it cannot directly explain their transcriptional state in *A. pisum*.

## Results and Discussion

### Young Paralogs in *A. pisum* Are under Neofunctionalization and Involve Diverse Biological Functions

**Fig. 1.**
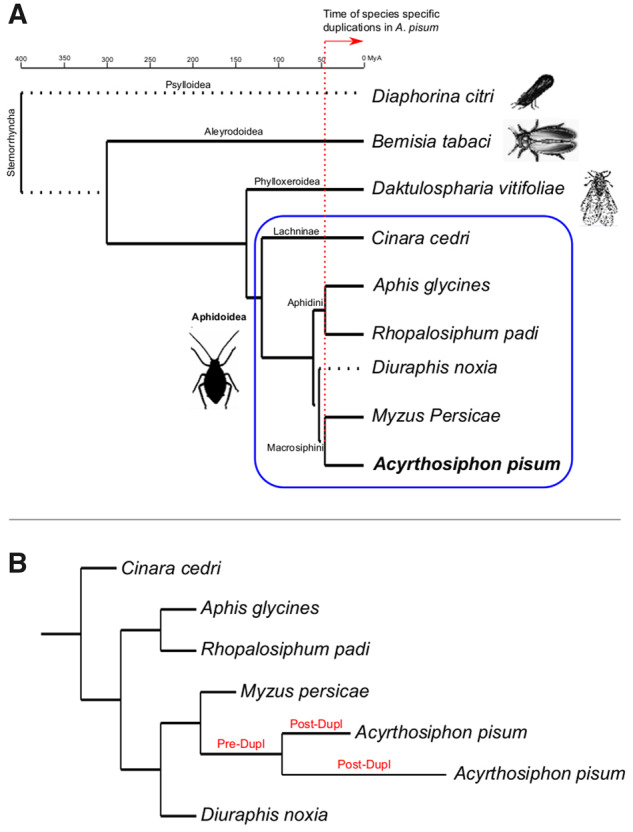
(*A*) Chronogram of Sternorrhyncha interrelationships. Systematic classifications (superfamily, family, subfamily, and tribe) are shown in each node/branch. Images selected from PhyloPic. Divergence times taken from TimeTree (Kumar et al. 2017). Dotted branches represent lineages for which divergence times were not available. (*B*) Example of individual gene tree showing a duplication in *Acyrthosiphon pisum*, as the genes selected from the present study (see Materials and Methods). Pre- and post-duplication branches as defined for the positive selection analysis are highlighted in red.

We built a phylome (i.e., the complete collection of phylogenetic trees for each gene encoded in a genome) for *A. pisum* in the context of hemipteran evolution, including five additional aphid species and three basal Sternorrhyncha species (fig. 1*A*). The phylome was then scanned for the presence of species-specific duplications. A total of 5,300 species/specific duplication events were detected in the *A. pisum* phylome that were clustered in 1,834 paralogous families. Due to the complexity of analyzing and interpreting highly expanded and old duplications, we divided the duplications into two different sets. The first set consisted of pairs of genes specifically duplicated in *A. pisum* which fulfilled the following criteria: 1) they presented a single-copy ortholog in at least two of the other species in the phylome, 2) the node where the two sequences duplicated in the tree was well supported, and 3) the tree reconstructed only using the selected paralogs and orthologs had to retain the duplication event. A second data set focused on genes that were duplicated more than once specifically in *A. pisum*. Since our goal was to understand the evolution of recent duplicates, in the case of these larger gene family expansions only pairs of genes found at the tips were considered. These pairs also had to fulfill the previous requirements. A total of 843 duplication events containing 1,686 genes were further analyzed. Among those, 606 came from single duplication events (the first set), whereas the remaining 237 were extracted from larger duplication events (from the second set; see supplementary table S3, Supplementary Material online, for the complete list of selected genes and families). Note also that in some cases (8.5% of cases), an ortholog to the closest relative to *A. pisum* (*Myzus persicae*) was missing, and hence, there is the possibility that these particular duplications are older.

**Fig. 2.**
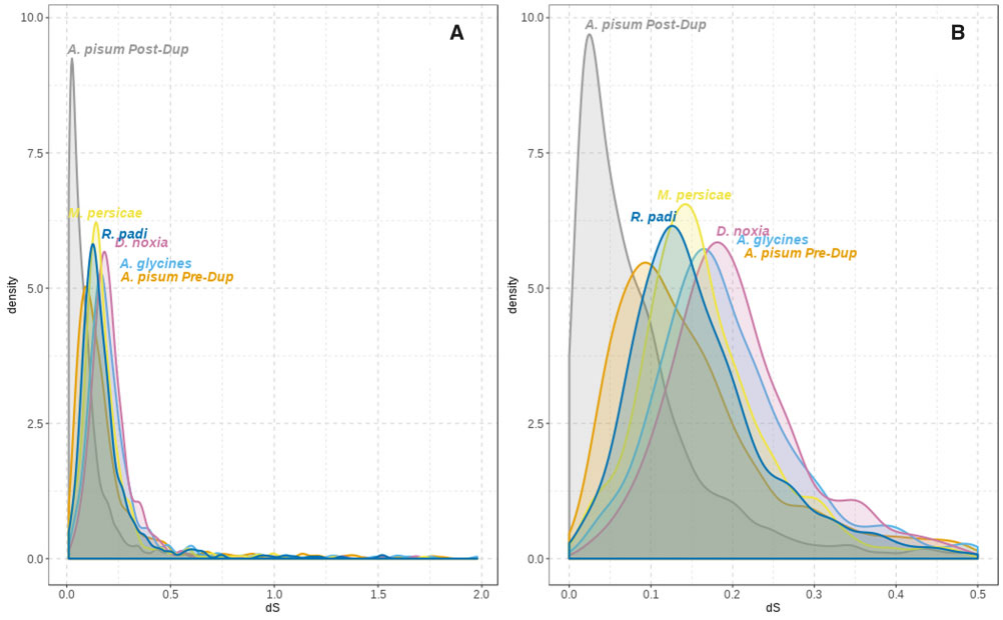
d*S* values for the selected duplications after filtering genes with d*S* > 2 and d*S* < 0.01 (*A*) and zoom by limiting *x*-axis to 0.5 (*B*). *Cinara cedri* was removed from this plot for visualization. d*S* for *Acyrthosiphon pisum* was calculated before (*A. pisum* Pre-Dup) and after (*A. pisum* Post-Dup) the duplication took place. See figure 1*B* for “Pre-Dup” and “Post-Dup” explanation.

We calculated the relative age of the selected duplications using the number of synonymous substitutions per synonymous site (d*S*) as a proxy. We estimated d*S* for each internal and terminal branch of each gene tree using the “free ratio branch model” from codeML, and we filtered out specific genes with d*S* > 2 and d*S* < 0.01 (see Materials and Methods for details). By comparing the distribution d*S* in each copy of the selected duplications (*A. pisum* Post-Dup) with the preduplication branches (*A. pisum* Pre-Dup) and single-copy orthologs (fig. 1*B*), we showed that lineage-specific genes in *A. pisum* are enriched in recent duplications represented by their low d*S* values compared with other species (fig. 2). Since it is known that gene conversion (GC) may decrease the divergence between paralogs, we scanned the respective coding sequences for the presence of GC tracts using GENECONV software (Sawyer 1989). We detected that 187 duplications (22.2%) showed evidence of a GC event between the two *A. pisum* sequences that remained significant after multiple-comparison correction. From those, 27 occurred in tandem duplicates (28.7% of total duplications in tandem), 31 in duplications in the same contig (31%) and 117 in duplications in different contigs (19.3%). Thus, tandem duplicates do not seem to be particularly enriched in GC; however, we cannot rule out that some of the selected duplicates may be older than inferred due to GC. In addition, due to the fragmentation of the genome assembly used, it is possible that some tandem duplicates are not detected in our analyses. In an initial characterization of our set of recent gene duplications, we estimated the median identity for each protein sequence of each gene family alignment using trimAl v1.3. We observed that *A. pisum* duplicates were significantly less similar at the sequence level between them than when compared with single-copy orthologs, suggesting that their sequences are diverging faster (supplementary fig. S1*A*, Supplementary Material online). Thus, despite the presence of GC tracts, this process was not enough to homogenize the sequence of the gene duplicates. As a consequence, we did not discard GC tracts from the sequences because, according to the literature, DNA sequences are useful to detect the presence of GC but not to correctly infer their length (Mansai et al. 2011).

**Fig. 3.**
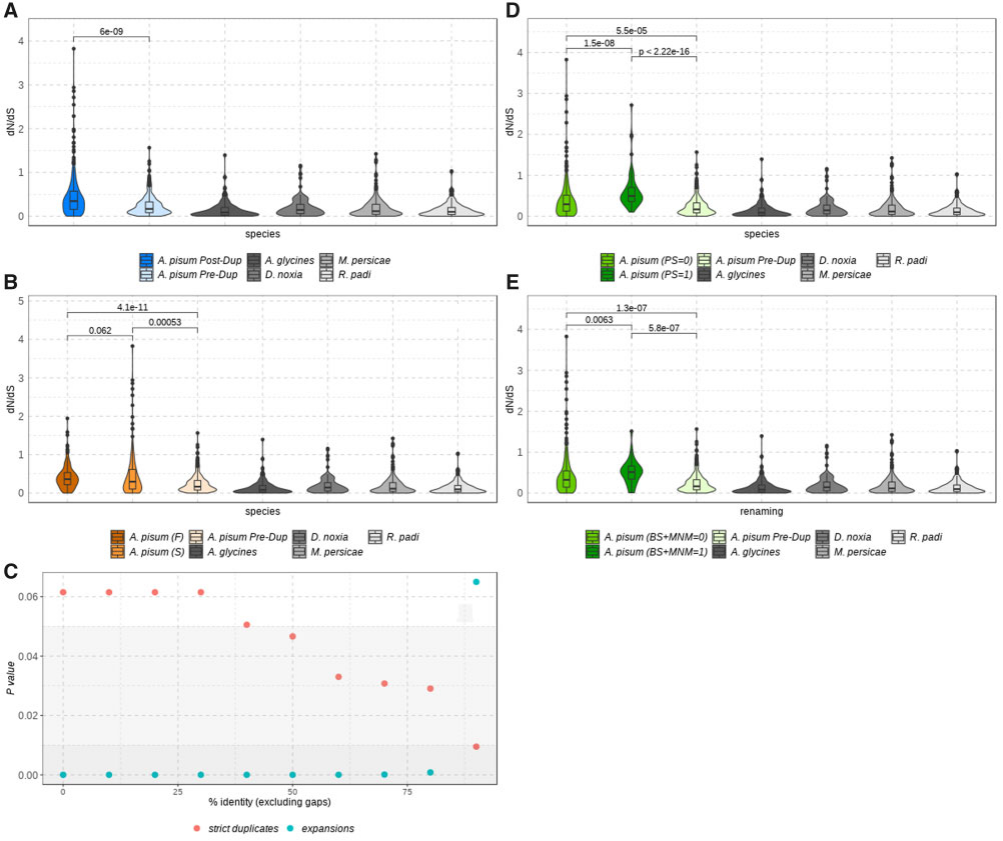
Evolutionary rate (d*N*/d*S*) for the selected duplications after filtering duplicates with d*S* > 2 and d*S* < 0.01 in any of the genes (201 strict duplicates remained). *Acyrthosiphon pisum* genes are colored, whereas single-copy orthologs are shown in gray scale. (*A*) *A. pisum* preduplication (Pre-Dup) and postduplication (Post-Dup) branches are shown. (*B*) *A. pisum* postduplication branches were classified as Fast (F) or Slow (S) according to d*S*. (*C*) *P* values comparing Fast (F) or Slow (S) copies from duplicates binned according to their percentage of identity (increasing by 10 the percentage of identity between bins). Background was colored according to the *P* value: dark gray for *P* value < 0.01, light gray for *P* value > 0.01 and <0.05, and white for *P* value > 0.05. (*D*) *A. pisum* postduplication branches were classified as having (PS = 1) or not (PS = 0) signals of positive selection. (*E*) *A. pisum* postduplication branches were classified as having (BS + MNM = 1) or not (BS + MNM = 0) signals of positive selection after BS + MNM model. *P* values for all plots were estimated using wilcox.test function from R.

To test the hypothesis that recently duplicated genes evolved faster and to evaluate the pace of evolution in our set of recent gene duplications, we calculated evolutionary rates as the ratio of nonsynonymous to synonymous substitution rates (d*N*/d*S*) for each gene family using codeML software from PAML package v4.9 (see Materials and Methods for details). This software computes individual estimations for each branch of a given tree, allowing us to calculate d*N*/d*S* before and after the duplication (hereafter called as preduplication [Pre-Dup] and postduplication [Post-Dup] branches, see fig. 1*B*). To facilitate the interpretation of the results, gene duplicates were divided into two groups: “strict duplicates” (i.e., genes with only two copies, ∼72% of the selected duplicates) and “expansions” (genes with more than two copies, see Materials and Methods for details). Paralogs of both “strict duplicates” and “expansions” had significantly faster rates as compared with their preduplicated ancestors as well as to single-copy orthologs (fig. 3*A* and supplementary fig. S2, Supplementary Material online). We then classified paralogous copies of each duplicated gene pair into “fast” and “slow” evolving copies, according to the d*S* values of the branch subtending each copy (see Materials and Methods), which allows to distinguish between subfunctionalization and neofunctionalization scenarios (Sandve et al. 2018). d*N*/d*S* was not homogeneous in the two copies, since the fast postduplication copy is evolving more rapidly than both the slow postduplication copy and the preduplication ancestor in the “strict duplicates” subset (fig. 3*B*). The statistical significance of these differences in d*N*/d*S* values depends on the percentage of identity between duplicate copies (fig. 3*D*). The “expansions” subset showed the opposite pattern, in which the slow copy had a higher d*N*/d*S* ratio than the fast one (supplementary fig. S2*B*, Supplementary Material online). It is important to highlight that expansions are complex families more prone to include missannotated genes or pseudogenes, which may influence our results. Thus, conclusions from this subset should be taken with caution. Asymmetrical evolution of gene duplicates has been observed in several organisms, such as fungi, *Drosophila melanogaster*, *Caenorhabditis elegans*, and human, which was attributed to relaxed selective constraints and, in some cases, to the action of adaptive selection (Conant 2003; Zhang 2005; Scannell and Wolfe 2007; Pegueroles et al. 2013; Pich I Roselló and Kondrashov 2014). To further evaluate whether asymmetrical evolution may be related to positive selection, we tested for positive selection using codeML (see Materials and Methods for details). We detected positive selection in 388 genes distributed in 316 duplications (supplementary tables S3 and S4, Supplementary Material online), which supports that positive selection contributed to the acceleration of a substantial fraction of duplicates (at least ∼37%). In addition, in most duplications, only one duplicate was under positive selection, with some exceptions where both duplicates showed signs of selection (28 and 44 for “strict duplicates” and “expansions,” respectively, supplementary table S4, Supplementary Material online). Interestingly, postduplication branches under positive selection have significantly different (and faster) rates (i.e., d*N*/d*S* values) than both the postduplication branch without positive selection and the preduplication branch in both subsets (fig. 3*C*), which is in agreement with the lower levels of identity detected for branches under positive selection (supplementary fig. S1*B*, Supplementary Material online, yellow boxplot). It is worth noting that our estimate of positive selection cases may be conservative due to the strict applied filtering and the inherent difficulty of detecting positive selection since this often acts during short periods of evolutionary time (Zhang 2005; Pegueroles et al. 2013; Pich I Roselló and Kondrashov 2014). However, a recent paper showed that the branch-site test cannot distinguish which sequence patterns have been caused by positive selection or by the neutral fixation of nonsynonymous multinucleotide mutations (MNMs) (Venkat et al. 2018). To evaluate this, we identified MNMs in our gene duplicates. First, we reconstructed the ancestral sequence using codeML (see Materials and Methods for details), and then we compared the derived and ancestral sequences codon by codon and counted the number of changes. Those codons having more than one change were considered as MNMs. Supplementary figure S3, Supplementary Material online, shows the percentage of MNMs per sequence for the duplicated genes, classified as having or not signals of positive selection. We observed that genes under positive selection were enriched in MNMs in both subsets. In order to test whether MNMs were biasing our estimates of positively selected genes, we evaluated the detection of genes under positive selection using a model that accounts for the presence of MNMs (BS + MNM model; see Materials and Methods). The new analysis recovered 155 genes under selection under the BS + MNM (supplementary fig. S3, Supplementary Material online). Therefore, ∼40% of the genes originally inferred as positively selected still maintained the signal of positive selection after accounting for MNMs. These genes thus represent a conservative set of genes under positive selection in the pea aphid. However, it should be noted that MNM-bearing genes can indeed be truly under positive selection, and as such we did not exclude them from our discussion.

**Fig. 4.**
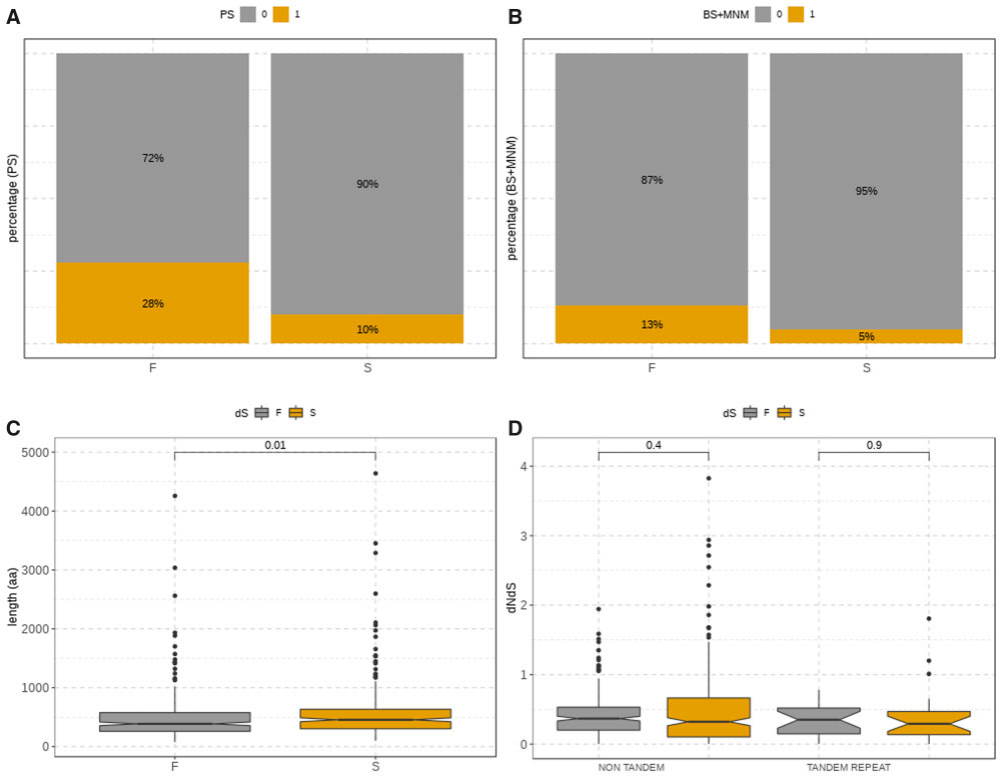
*Acyrthosiphon pisum* genes from the selected duplications classified as Fast (F) or Slow (S) according to d*S*, after filtering duplications with d*S* > 2 and d*S* < 0.01 (201 strict duplicates remained, see Materials and Methods for details). (*A*) Percentage of genes under positive selection (PS = 1 in ochre) or with no signal of positive selection (PS = 0 in gray); (*B*) percentage of genes positively selected after BS + MNM model (BS + MNM = 1 in ochre) or not (BS + MNM = 0 in gray); (*C*) cDNA length (in aa); and (*D*) d*N*/d*S* after classifying duplicates according to their relative location (i.e., tandem and nontandem duplicates). *P* values were estimated using wilcox.test function from R.

To evaluate the impact of positive selection in pairs of gene duplicates, we discarded the duplicates with d*S* > 2 or d*S* < 0.01 in any of the duplicated genes (fig. 4*A* and supplementary fig. S4, Supplementary Material online). The fraction of duplicates under positive selection is higher for the fast paralogs as compared with their slow counterparts (fig. 4*A* and supplementary fig. S4, Supplementary Material online) which supports that the asymmetrical increase in rates may be due to adaptive selection, at least in a fraction of the duplications analyzed, especially in the “strict duplicates” subset. We also observed that the fast postduplication copies tend to have shorter sequence lengths in the “strict duplicates” subset (fig. 4*B*, not in the “expansions” subset as shown in supplementary fig. S4*B*, Supplementary Material online). d*N*/d*S* values of the fast and slow evolving copies were similar independently of being consecutively positioned in the genome or not, particularly in the “strict duplicates” set (fig. 4*C*). It is worth noting that there are more cases of positive selection in the “expansions” subset, and consequently positive selection is more prone to be wrongly assigned to this subset due to its complexity.

We further evaluated the presence of selective pressures on *A. pisum* genome by estimating Tajima’s *D* for all annotated genes. We obtained variation data from three pea aphid host races specialized on different crops: alfalfa, pea, and genista (Grigorescu et al. 2018; Nouhaud et al. 2018). We observed differences in Tajima’s *D* values between races, but overall values are negative in the three of them (supplementary figs. S5–S8, Supplementary Material online). Negative values indicate an excess of rare alleles, which may be due to purifying selection or a recent population expansion after a bottleneck. We splitted the genome into “strict duplicates,” “expansions,” and the rest of the genes (which are mostly not duplicated) and estimated Tajima’s *D* in these subsets of genes. We observed the same trend in the three host races. When considering all genes as a whole, “expansions” have significantly higher values than both “strict duplicates” and the rest of the genes (with the only exception of genista population), which may indicate that the selective pressure to constrain “expansions” is lower (supplementary fig. S5, Supplementary Material online). However, no significant differences were found when comparing Tajima’s *D* for the fast and slow evolving copies in none of the subsets, indicating that both copies may have undergone similar levels of purifying selection. Remarkably, we observed that exons have significantly lower values compared with introns. Thus, negative values are more likely to be driven by purifying selection rather than a recent population expansion, even if changes in population size are expected in a species that reproduces by cyclical parthenogenesis, as is the case in *A. pisum*. We also investigated codon usage index in gene duplicates, since departures from optimal codons in one of the copies would suggest relaxed purifying selection. Most of the duplicates (62%) have an optimal codon usage, whereas in 32% of the cases both copies have a nonoptimal codon usage. For pairs of duplicates in which one of the copies was under positive selection, we further explored whether there were differences in optimal codon usage. Out of the 244 duplicates in which only one gene is under positive selection in the pair of duplicates, only nine pairs showed a low codon adaptation index in the positively selected gene. A similar number (eight pairs) was recovered for the opposite scenario (i.e., in pairs of genes where the gene under selection was the one showing a better codon usage). Thus, our set of recent duplicates does not seem to have a differential relaxation of purifying selection among copies. Altogether, these observations suggest that, at least for the “strict duplicates” subset, neofunctionalization fueled by positive selection is the most likely scenario underlying the observed evolution patterns of recent duplicates in *A. pisum*.

To identify the putative functions of the duplicated and positively selected genes, we tested whether the resulting paralogs were enriched in any particular functions through Gene Ontology (GO) enrichment analyses. We explored enrichment only in strict duplicates as described above, which accounts for 72% of all duplicates. Similar results including enrichment in neurotransmitter metabolism, neural retina development, biosynthesis, and metabolism of glutamate, quinone, and ammonia (supplementary fig. S9, Supplementary Material online) were found when the list of strict duplicates under selection was compared with all the genome or with the complementary portion of the genome (i.e., all genes but the pairs of strict duplicates where at least one gene is under positive selection). This suggests that neofunctionalization may be affecting all these functions. Strict duplicates under positive selection did not result in any functions enriched when compared with all strict duplicates.

A first example of a gene positively selected that may have undergone neofunctionalization is the gene encoding the protein *maelstrom 2* (UniProtKB B3MZY6 MAEL2_DROAN), that in *Drosophila ananassae* has been predicted to play a central role during oogenesis by repressing transposable elements and preventing their mobilization, essential for maintaining the germline integrity (Sato et al. 2011). It is also the case of the genes encoding for glutamine synthetase 2 (UniProtKB J9JML2_ACYPI) and other genes involved in glutamate metabolism such as the genes encoding aspartate aminotransferase 2 (UniProtKB J9JIS1_ACYPI), glutamate dehydrogenase (UniProtKB J9KB74_ACYPI), and glutamate decarboxylase (UniProtKB DCE_DROME). These genes belong to the same pathway of incorporation of ammonium nitrogen into glutamate cycle to assimilate ammonia into glutamate: Those genes have been shown to be upregulated in bacteriocytes in *A. pisum*, which function as specialized symbiont-bearing organs of amino acid production (Hansen and Moran 2011). Therefore, positive selection and neofunctionalization may have facilitated host–symbiont cooperation in the production of amino acids between the pea aphid and *Buchnera*, such as for the amino acid transporters as shown by Duncan et al. (2016). Overall, these two examples illustrate how key biological functions (oo- and morpho-genesis and host–symbiont cooperation) might have been reshaped through duplication followed by neofunctionalization in the pea aphid.

### Tissue Divergence Patterns in Duplicated Genes Range from Low to High

We have shown that recent *A. pisum* duplicates have different evolutionary rates (using d*N*/d*S* values as a proxy) and that a substantial fraction of them are evolving under positive selective pressure. The different behavior of the two copies may result in differences in gene expression levels. To evaluate this hypothesis, we compiled RNA-Seq data from a total of 106 libraries grouped into 18 different conditions (see Materials and Methods). A principal component analysis showed that samples are clustered by tissues when considering all annotated genes in the *A. pisum* genome as well as when subtracting the genes of the “strict duplicates” and “expansions” subsets (supplementary fig. S10, Supplementary Material online). To compare gene expression profiles across tissues, we profiled the expression of each selected gene, being 0 for not expressed and 1 for expressed (supplementary table S5, Supplementary Material online, see Materials and Methods for details). Interestingly, we observed that 329 duplicates (i.e., ∼39% of the 843 selected duplicates, 212 of them “strict duplicates” and 117 “expansions”) showed differences in their tissue expression pattern in at least one tissue. When considering their location, we observed that corresponds to 40.4% of the 705 DNA-based pairs (i.e., duplicated by copying and pasting a DNA sequence from one genomic region to another), 36.4% of the 44 retrocopies (i.e., mRNA-mediated gene duplicates), and 29.8% of the 94 tandem duplicates (i.e., duplicated one after the other in the genome). Thus, most tandem duplicates (∼70%) tend to have the same expression patterns, as expected (Lercher et al. 2003). In order to measure the expression divergence between duplicates in the 18 conditions, we computed three different statistics using a binary profiling binning approach: hamming distance, tissue expression complementarity distance and tissue divergence (d*T*, supplementary table S4, Supplementary Material online, see Materials and Methods for details). The three methods show similar results in both subsets (“strict duplicates” and “expansions”), which is in agreement with the high correlation between them (supplementary fig. S11, Supplementary Material online). Overall, tissue divergence between duplicates is low, with mean values ranging from 0.09 to 0.20 and the median being 0 in the three methods, which was expected since ∼71% of the duplicates have the same expression profile. As expected, when considering merely pairs with differences in the expression profile we obtained higher values (median values ranged from 0.33 to 0.20). Interestingly, the maximum value detected is 1 for the three methods, meaning that some pairs of duplicates have totally opposite expression patterns.

**Fig. 5.**
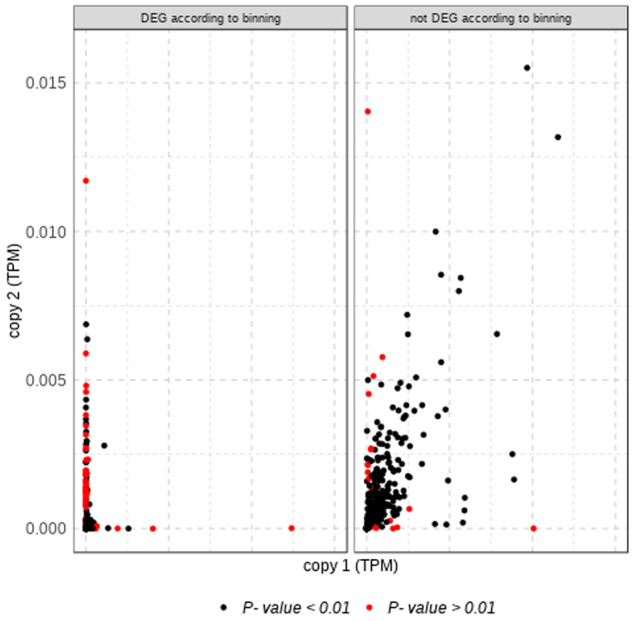
Scatterplot showing median gene expression between pairs of duplicates for the “strict duplications” subset. Colors correspond to the *P* values of a Pearson correlation (see Materials and Methods for details). The significance of the test should be interpreted with caution for those genes that are lowly expressed (i.e., those located in the lower left corner). For visualization purposes, we discarded five gene pairs that were outliers in the subset “not DEG according to binning.”

We also compared gene expression between copies estimated as transcripts per million (TPMs). It is worth noting that overall gene expression values for “expansions” is significantly lower than for “strict duplicates” (median values were 1.07e-04 and 2.43e-05, respectively, *P* value = 2.64e-07). This may reflect real biological differences such as higher tissue specificity or lower expression of genes that are part of large family expansions, although it may also be due to the difficulty to assign gene expression to a given copy in these complex expansions and/or to the likely higher amount of missanotations or pseudogenes in this subset, as discussed above. We compared gene expression across tissues, by computing Pearson correlations and building linear models within gene duplicates (see Materials and Methods for details). If the two copies have similar expression patterns across tissues we should expect high Pearson correlations and *r*^2^ values. Overall, our findings are in line with the binning approach, since both Pearson correlations and *r*^2^ values are high (0.91 and 0.82, respectively, for “strict duplications” and 0.77 and 0.60 for “expansions”; supplementary table S6, Supplementary Material online). In addition, the subset of differentially expressed genes obtained in the binning analysis is enriched in differentially expressed genes according to our models (fig. 5 and supplementary fig. S12, Supplementary Material online). Thus, we can conclude that the two subsets tend to have similar expression patterns in the two copies, despite the fact that there are some interesting differences as we will discuss below.

### Positive Selection May Modulate Differences in Gene Expression

Positive selection might be correlated with sub- or neo-functionalization by acquiring a new expression profile. To test this hypothesis, we compared the tissue expression patterns between gene duplicates. We found different expression patterns in 50% of pairs with two copies under selection (35.7% of 14 “strict duplicates” and 59% of “expansions”), 36.9% of pairs with only one copy under selection (30% of “strict duplicates” and 50.7%of “expansions”), and 39.6% of pairs with no copy under selection (37% of “strict duplicates” and 47.2% of “expansions”). This suggests that positive selection plays a role in gene transcription regulation but other factors are also involved, since in the absence of positive selection, differences in gene expression were also detected. When focusing on duplications that have different expression patterns in at least one of the studied conditions, we observed that tissue expression divergence levels were similar for duplications having or not copies under selection in both subsets (“strict duplicates” and “expansions”) as well as overall duplicates (supplementary figs. S13 and S14, Supplementary Material online). For the 244 duplications with positive selection in one copy, we quantified the cases in which a gene expression was gained or lost in any of the tissues considering the expression profile of the copy with absence of positive selection as background (supplementary table S5, Supplementary Material online). The number of losses was higher than that of gains in the “strict duplicates” subset (40 and 15, respectively), meaning that in most cases the gene expression profile of the copy under selection is reduced as compared with the nonselected copy. In other words, the selected copy is expressed in a subset of tissues at least in the “strict duplicates” subset, since the median number of tissues in which the nonselected and the selected copies are expressed is 11 and 8.5, respectively, in this set of duplicates. In the “expansions” subset the amount of gains and losses is quite similar (21 and 19, respectively), as well as the median number of tissues in which the nonselected and the selected copies are expressed (9 and 10, respectively). The pattern observed in the “strict duplicates” subset is consistent with a specialization scenario, in which one copy is expressed in all (or most) tissues but at least one copy is not. This scenario, which can be considered a particular case of subfunctionalization, has been proposed to be the main fate after whole genome duplication (Marlétaz et al. 2018) and may influence the evolution of young duplicates (Huerta-Cepas, Dopazo, et al. 2011). In addition, the 15 cases in which the selected copy is expressed in at least a tissue in which the nonselected copy has no expression (gain cases) are candidates that may have undercome neofunctionalization after gene duplication (table 1). From these 15 duplications, 9 showed similar predicted annotation between the pairs (duplications 288, 322, 397, 460, 489, 576, 633, 795, and 840). The six other duplications include pairs with different predicted annotations. For the 15 duplications (i.e., 30 genes), 16 genes are uncharacterized with no predicted functions, 5 are annotated as zinc finger putative proteins, and 3 are dynein-like proteins.

**Table 1.** Duplications in Which the Copy under Positive Selection (PS = 1) Is Expressed in at Least One Tissue in Which the Nonselected Copy (PS = 0) Has No Expression (highlighted in dark gray).

Duplication Name	Gene Code	Putative Function	PS	BS-M	AM	AP	AO	E0	E1A	E1K	E2A	E2K	E3A	E3K	H	HP	HR2	HR4	LP	G	SG	B
duplication_159	LOC103311559	Uncharacterized	0	0	0	0	0	0	0	0	0	0	0	0	NA	0	0	0	0	0	NA	0
duplication_159	LOC100166252	PiggyBac transposable element-derived protein 4-like	1	0	NA	NA	NA	NA	NA	NA	NA	NA	1	1	NA	NA	0	0	0	1	NA	NA
duplication_213	LOC100570324	Uncharacterized	0	0	1	0	NA	NA	1	1	NA	1	NA	NA	0	1	NA	0	0	NA	NA	NA
duplication_213	LOC100574933	PAX-interacting protein 1-like	1	0	1	1	1	1	1	1	1	1	1	1	NA	NA	1	1	1	NA	1	1
duplication_288	LOC100572588	Uncharacterized	0	1	1	1	1	1	1	1	1	1	1	1	1	1	0	0	1	1	1	1
duplication_288	LOC103308356	Uncharacterized	1	0	0	1	1	1	1	1	1	1	1	1	1	1	1	1	NA	1	1	1
duplication_322	LOC107883251	Uncharacterized	0	0	0	NA	NA	1	NA	1	1	1	1	1	1	NA	1	1	NA	1	1	1
duplication_322	LOC107883068	Uncharacterized	1	1	1	1	NA	1	1	1	1	1	1	NA	1	1	1	1	1	1	1	1
duplication_340	LOC103310866	Uncharacterized RING finger protein C32D5.10-like	0	0	0	0	0	NA	0	0	0	0	0	0	0	0	0	0	0	NA	0	0
duplication_340	LOC100571229	E3 ubiquitin-protein ligase Topors-like	1	0	1	1	1	1	1	1	1	1	1	1	0	NA	NA	0	1	NA	NA	1
duplication_397	LOC100162340	Dynein heavy chain 1, axonemal	0	0	1	0	0	0	0	0	0	NA	0	0	0	0	0	NA	0	NA	0	0
duplication_397	LOC107882216	Dynein heavy chain 1, axonemal-like	1	0	0	NA	1	0	0	0	NA	0	NA	0	NA	1	0	NA	0	NA	NA	NA
duplication_4	LOC100568916	Uncharacterized	0	0	1	NA	1	1	1	1	1	1	1	1	NA	NA	0	0	NA	NA	NA	NA
duplication_4	LOC100160128	26S proteasome non-ATPase regulatory subunit 12-like	1	0	1	1	1	1	1	1	1	1	1	1	NA	1	1	1	1	NA	NA	1
duplication_460	LOC107882168	Zinc finger protein 134-like	0	0	0	0	NA	0	NA	NA	0	0	NA	NA	0	0	0	0	0	NA	NA	0
duplication_460	LOC103310004	Zinc finger protein 134-like	1	0	1	0	1	NA	NA	0	0	NA	0	0	0	0	0	0	0	NA	NA	0
duplication_489	LOC107882711	Uncharacterized	0	0	NA	1	1	1	1	1	1	1	1	1	NA	1	1	1	0	1	NA	1
duplication_489	LOC100162929	Uncharacterized	1	0	1	1	1	1	1	1	1	1	1	1	1	1	1	1	1	1	1	1
duplication_576	LOC100569858	Uncharacterized	0	0	1	0	0	NA	NA	0	0	0	0	0	NA	0	0	0	0	NA	NA	0
duplication_576	LOC100574180	Uncharacterized	1	0	1	0	0	NA	NA	0	NA	NA	0	NA	0	NA	1	NA	0	1	1	0
duplication_633	LOC103309550	Zinc finger MYM-type protein 1-like	0	0	0	0	0	0	0	0	0	0	0	0	NA	0	0	0	0	NA	NA	0
duplication_633	LOC103307955	Zinc finger MYM-type protein 1-like	1	0	0	NA	NA	0	NA	0	0	NA	0	0	NA	NA	NA	0	0	NA	NA	1
duplication_663	LOC107884962	Uncharacterized	0	0	0	0	0	1	NA	1	NA	NA	NA	1	NA	0	0	0	0	1	1	NA
duplication_663	LOC100570263	Uncharacterized	1	0	NA	1	1	1	1	1	1	1	1	1	NA	1	NA	0	1	NA	1	1
duplication_693	LOC100165085	Uncharacterized	0	0	1	0	0	NA	0	NA	0	NA	0	0	NA	0	NA	NA	NA	NA	NA	1
duplication_693	LOC100165046	Dynein heavy chain 7, axonemal-like	1	1	NA	1	0	1	1	1	1	1	1	1	1	1	1	1	1	1	1	1
duplication_795	LOC107882727	Sialin-like	0	0	0	NA	0	0	0	0	0	0	0	0	NA	1	1	1	1	NA	NA	NA
duplication_795	LOC100164217	Putative inorganic phosphate cotransporter	1	0	1	1	1	NA	1	1	1	1	1	1	1	1	1	1	1	1	1	1
duplication_840	LOC100571978	Uncharacterized	0	0	1	0	0	0	0	0	0	0	0	0	0	0	0	0	0	0	NA	0
duplication_840	LOC107884413	Uncharacterized	1	0	0	0	0	0	NA	NA	0	NA	NA	0	NA	NA	1	1	0	NA	NA	0

Note.—NA, no information on gene expression available (see Materials and Methods for further details about each condition type); PS, positive selection; BS-M, branch-site test model accounting for MNM; AM, adult males; AP, adult females parthenogenetic; AO, adult female oviparae; E0, embryos stage 17; E1A, embryos stage 18 sex; E1K, embryos stage 18 asex; E2A, embryos stage 19 sex; E2K, embryos stage 19 asex; E3A, embryos stage 20 sex; E3K, embryos stage 20 asex; H, Head_Mix; HP, head adult female parthenogenetic; HR2, head larvae 2; HR4, head larvae 4; LP, legs adult female parthenogenetic; G, gut; SG, salivary glands; B, bacteriocyte.

### Chromatin Accessibility Is Altered in Young Duplicated Genes but Does Not Correlate to Gene Expression

RNA-Seq and FAIRE-Seq data were analyzed together for each of the predicted genes in the *A. pisum* genome (supplementary table S7, Supplementary Material online). FAIRE-Seq is a molecular technique that allows to detect nucleosome-depleted regions of the genome, which are usually found in open chromatin. We validated the overall correlation of transcription and chromatin accessibility (supplementary fig. S15, Supplementary Material online, for embryos and Richard et al. 2017, fig. 5 for whole body of males and females), ensuring the quality of the data sets. In order to explore the correlation between transcriptional status and chromatin accessibility, genes were classified in four categories depending on their expression and TSS log 2 (FAIRE/Input) values (see Materials and Methods): 1) open and expressed, 2) open and not expressed, 3) closed and expressed, and 4) closed and not expressed. Concerning all genes, we found that most (8,870 genes: 64%) belonged to the category “closed and expressed,” which could potentially be due to the threshold applied to define “open genes” and to the higher sensitivity of RNA-Seq compared with FAIRE-Seq. From the remaining, 18% of the genes were “open and expressed,” and the other 18% “closed and not expressed” (2,497 and 2,491 genes, respectively). The “open and not expressed” category was barely represented in the data set (0.3% of the assigned genes). This result reflects the quality of the FAIRE-Seq data processing since it is expected that “open and not expressed” genes are virtually absent as they will violate the common rules of gene transcription, therefore corresponding to false positives. GO term enrichment analysis for each of the four categories revealed that genes “open and expressed” were enriched in transcription factor activity (GO:0003700, molecular function) and sequence-specific DNA binding (GO:0043565, molecular function). Genes “closed and not expressed” were enriched in nucleic acid binding (GO:0003676, molecular function). The two remaining categories were not significantly enriched in any functions, despite the high number of genes in the “closed and expressed” category: This highlights the biological relevance of gene classes displaying coordinated expression and TSS accessibility in this data set.

Regarding the set of young gene duplicates, we found that 26% of them had different chromatin states in each paralog (i.e., one paralog was detected as open chromatin and the other as closed) (173 out of 666 duplicates, supplementary table S8, Supplementary Material online; see some examples in supplementary fig. S16, Supplementary Material online). If we divide young duplicates into “strict duplicates” and “expansions” we observed similar patterns (26% and 27%, respectively). To test whether different expression patterns correlated to different chromatin states in each paralog, we searched for duplicates where each paralog belonged to a different category (i.e., categories [1]–[4] combining chromatin state and expression pattern, as described above) for the embryos and adult morphs, conditions for which FAIRE-Seq data were available (supplementary table S8, Supplementary Material online). Seventy-three pairs of duplicated genes did belong to different categories. From those, in 54 pairs both duplicates were expressed, with one copy being open and the other closed. In 19 pairs, both duplicates were closed but one gene was expressed and the other one not.

Our results indicate that the number of genes with closed chromatin (*n* = 11,322) was higher than for open chromatin (*n* = 2,536). This reduces overall the possible correlation of genes expressed and accessible at the same time, consequently hampering any gene-by-gene comparisons such as in the case of duplicated genes. Our results are in line with those of the integration of RNA-Seq and ATAC-Seq (similar to FAIRE-Seq) performed in a study in human tissues (Ackermann et al. 2016). They discussed that the poor correlation between RNA-Seq and ATAC-Seq data may be due to gene activation depending on multiple regulatory regions, possibly being located far from the gene locus itself. Indeed, FAIRE-Seq in whole body individuals or embryos is far less precise than at the level of cells or tissues, thus making the correlation between expression and accessibility even trickier. Also, since FAIRE-Seq only allows to test for *cis*-regulatory interactions, it may be hypothesized that most of the genes may be *trans*-regulated, which was impossible to determine with the data at hand considering the noncompleteness of the pea aphid genome assembly. Moreover, our analyses were centered on putative TSS which have not been validated experimentally. Nevertheless, we identified that the chromatin accessibility of the TSS of duplicated genes was different between the pairs in more than half of the cases. This shows that the chromatin state of promoters of simple pairs of duplicated genes can evolve independently in each member of the pair. A similar pattern has been observed in nematodes, where differential chromatin states were observed in new pairs of duplicated genes (Werner et al. 2018).

## Conclusions

Our study shows that recent simple gene duplicates—those formed by two duplicated genes—evolved asymmetrically in *A. pisum*, with one paralogous copy being more conserved and the other more divergent. The conserved copy was under the effect of purifying selection and hardly ever under the effect of positive selection. The divergent copy was usually positively selected and showed a faster evolutionary rate. Altogether, these results suggest that neofunctionalization may be one of the driving forces affecting young gene duplicates in *A. pisum.* In addition, genes under positive selection were putatively related to a large and diverse number of functions, indicating that neofunctionalization has a broad impact in multiple functions of the pea aphid biology. Remarkably, neofunctionalization may also be involved in symbiosis functioning, facilitating host–symbiont cooperation between *A. pisum* and *Buchnera*. More complex scenarios may have driven the evolution of large expansions, which remain difficult to analyze due to confounding factors, such as possible missannotations and pseudogenes. Concerning the expression patterns of the duplicated genes, we observed that more than one third of the duplicates showed different expression patterns, with some of them being under adaptive selection. This suggests that positive selection might not be the main or the only factor driving such differences in gene expression. For those duplicates with signals of positive selection, we found that a loss of function in a specific tissue is the most likely outcome, consistent with a scenario of tissue specialization and/or subfunctionalization. In contrast, we also found examples of genes under positive selection that gained their function in some tissues, compatible with a scenario of neofunctionalization.

Lastly, we did not find a relationship between chromatin accessibility and gene expression, which may potentially be explained by technical issues such as a limited prediction of TSS in the pea aphid genome coupled to the inherent low signal over background ratio of FAIRE-Seq data (Ackermann et al. 2016). Moreover, although this discrepancy may be due to the different sensitivity of both RNA-Seq and FAIRE-Seq, it may also reflect a pervasive level of *trans* regulation in the pea aphid genome (as seen in humans [Ackermann et al. 2016]). Nevertheless, we showed that more than half of the young duplicated genes selected had different chromatin states. This indicates that FAIRE-Seq technique is sensitive to differences in chromatin dynamics even in recent gene duplicates.

Altogether, our results indicate that gene duplication provided an arena of genetic novelty to reshape the genome of the pea aphid through positive selection, neofunctionalization, and tissue-specific expression in young duplicated species-specific genes. The relationships between these evolutionary scenarios are complex and difficult to disentangle. We emphasize that phylogenomic-centered studies are therefore most needed to further understand genome evolution in nonmodel organisms.

## Materials and Methods

### Identification and Selection of Duplications in the Pea Aphid Genome

The phylome (i.e., the complete collection of phylogenetic trees for each gene in its genome) of *A. pisum* Mordvilko, 1914 was reconstructed in the context of Hemiptera evolution. In addition to this species, belonging to the suborder Sternorrhyncha and to the family Aphididae and tribe Macrosiphini, we selected representatives of several hemipterans based on phylogenetic position and availability of a fully sequenced genome: *Diaphorina citri* Kuwayama, 1908 (Sternorrhyncha, Psylloidea), *Bemisia tabaci* (Gennadius, 1889) (Sternorrhyncha, Aleyrodoidea), *Daktulosphaira vitifoliae* (Fitch, 1855) (Sternorrhyncha, Phylloxeridae), *Cinara cedri* (Curtis, 1835) (Sternorrhyncha, Aphidoidea), *Diuraphis noxia* (Kurdjumov, 1913) (Sternorrhyncha, Aphidoidea), *Aphis glycines* (Matsumara, 1917) (Sternorrhyncha, Aphidoidea), *M. persica*e (Sulzer, 1776) (Sternorrhyncha, Aphidoidea), and *Rhopalosiphum padi* (Stal, Linnaeus, 1758) (Aphidinae, Aphidini) (fig. 1). Genome versions and number of predicted proteins are indicated in supplementary table S1, Supplementary Material online.

Phylomes were reconstructed using the PhylomeDB pipeline (Huerta-Cepas, Capella-Gutierrez, et al. 2011). For each protein encoded in the *A. pisum* genome, a BLAST search was performed against the custom proteome database built from the genomes listed above. Results were filtered using an *e*-value of 1e-05 and a minimum overlapping region of 0.5. Multiple sequence alignments were reconstructed in both directions using three different programs (MUSCLE v3.8 [Edgar 2004], MAFFT v6.712b [Katoh 2005], and KALIGN v2.04 [Lassmann and Sonnhammer 2005]) and combined using M-COFFEE (Wallace et al. 2006). A trimming step was performed using trimAl v1.3 (Capella-Gutierrez et al. 2009), consistency-score cutoff = 0.1667 and gap-score cutoff = 0.9. Following model selection, the best model in terms of likelihood as selected by the Akaike Information Criterion was chosen for tree reconstruction. Phylogenetic trees were inferred using PhyML v3.0 (Guindon et al. 2010). Four rate categories were used and invariant positions were inferred from the data. Branch support was computed using an approximate likelihood ratio test (aLRT) based on a chi-square distribution. Resulting trees and alignments are stored in PhylomeDB 4.0 (Huerta-Cepas et al. 2014, http://phylomedb.org). The phylomeID is 441 (http://phylomedb.org/phylome_441).

A species-overlap algorithm, as implemented in ETE v3 (Huerta-Cepas, Dopazo, et al. 2010; Huerta-Cepas, Marcet-Houben, et al. 2010), was used to infer orthology and paralogy relationships from the phylogenetic trees reconstructed in the phylome. The algorithm scans the tree and calls speciation or duplication events at internal nodes based on the presence of common species at both daughter partitions defined by the node. Gene gains and losses were calculated on this basis. Duplication ratios per node were calculated by dividing the number of duplications observed in each node by the total number of gene trees containing that node: theoretically, a value of 0 would indicate no duplication, a value of 1 an average of one duplication per gene in the genome, and >1 multiple duplications per gene and node.

To build the species tree, one-to-one orthologs present in all species were selected, resulting in a final alignment with 1,047 genes and 635,610 amino acid positions after concatenation. To ensure a congruent phylogenetic hypothesis under different models, a series of approaches were followed to infer the species tree. First, an maximum likelihood (ML) tree was reconstructed with PhyML under the best selected model of amino acid evolution (LG, Le et al. 2008). Second, a supertree was reconstructed using DupTree (Wehe et al. 2008) based on all the trees reconstructed in the phylome. Both phylogenies were congruent (fig. 1).

### Detection and Selection of Gene Duplications

For each gene tree, we first selected with ETE v3 (Huerta-Cepas, Dopazo, et al. 2010; Huerta-Cepas, Marcet-Houben, et al. 2010) the nodes that exclusively contained multiple *A. pisum* sequences. These were considered as species-specific duplications in *A. pisum*. Overlapping species-specific duplications were fused when they shared more than 50% of their members. Trees were then scanned for the presence of pairs of duplicates in *A. pisum* whose duplication node was highly supported (aLRT > 0.95) and which had at least two single-copy orthologs. Note that the selected pairs of duplicates are not limited to genes that duplicated just once, some of them belong to a (larger) expansion in which case the chosen pairs were always at the tips of the tree. Species-specific duplicated genes and selected orthologs were grouped and used to build a second ML tree. The purpose of this tree was to ensure that the resulting topology still contained the species-specific duplication. Pairs of duplicates with incongruent coding sequences annotation or unsatisfactory topology were discarded. This resulted in a final number of scrutinized duplications of 843. For each duplication, we obtained multiple protein sequence alignments with PASTA v1.8.3 (Mirarab et al. 2015). For each alignment, we computed a gene tree (using the tree-estimator RAxML option) that was used for the codeml analyses (see below) and we estimated the median identity score for each protein sequence in the alignment compared with all the others using trimAl v1.3 (-sident option; Capella-Gutierrez et al. 2009). We finally back-translated protein multiple sequence alignments into nucleotidic with trimAl (using -phylip_paml -nogaps -backtran options). GC was estimated from back-translated sequences using GENECONV software (Sawyer 1989), by considering fragments with evidence of a GC event between the ancestors of two *A. pisum* sequences that remained significant after multiple-comparison correction.

### d*N*/d*S* and Additional Filtering

We estimated the number of synonymous substitutions per synonymous site (d*S*), the number of nonsynonymous substitutions per nonsynonymous site (d*N*) and d*N*/d*S* ratio using the “free ratio branch model” implemented in codeML from PAML v. 4.9 (Yang 2007), using model = 1, CodonFreq = 3, and Nsites = 0 as options. This software allows to estimate d*S*, d*N*, and d*N*/d*S* for each internal and terminal branch of a given tree and also to reconstruct the ancestral sequence before the duplication occurred. Analyses were computed for the 1,020 selected duplications, which contained a specific duplication in *A. pisum* and at least two single-copy orthologs for any of the other eight species included in the phylome. We noticed that d*S* for the three more distant species was much higher (the percentage of sequences with d*S* > 2 was 82.1%, 86.2%, and 63.9% in *Diaphorina citri*, *Bemisia tabaci*, and *D. vitifoliae*, respectively) than in the closely related species (16.5%, 6.4%, 5.8%, 3.3%, 4.4%, and 0.7% in *C. cedri*, *Aphis glycines*, *Rhopalosiphum padi*, *Diuraphis noxia*, *M. persicae* and *A. pisum*, respectively). Since such large d*S* values may indicate problems in the orthology identification, we discarded duplications with only single-copy orthologs in the three most distant species. A total of 843 duplications remained after this filtering.

### Age of the Selected Duplications and Classification into Fast and Slow Copies

The relative age of the selected duplications was calculated using the number of synonymous substitutions per synonymous site (d*S*) as a proxy. From the total of 5,589 genes from six species in 843 duplications, we filtered out genes with d*S* > 2 (which may indicate problems in the orthology identification, 242 genes) and d*S* < 0.01 (which may lead to high d*N*/d*S* ratios with no biological sense, 908 genes). We also used the d*S* estimates to classify the two copies of each selected duplication into fast and slow, by comparing their d*S* values, the copy with the lowest d*S* value being classified as slow and the other as fast.

### Classification of Gene Duplications

Gene duplicates can be divided into two groups: strict duplicates (606), including *A. pisum* genes that derive from a recent common ancestor and duplicated specifically in this species only once (i.e., genes with only two copies) and expansions (237), including *A. pisum* genes that also derive from a recent common ancestor but duplicated multiple times (i.e., genes with more than two copies). In addition, duplicates were further classified as tandem (defined as duplicates with no genes in between; 94 in total), located in the same or different contig (dispersed duplicates, 100 and 605, respectively) and retrotransposed (defined as dispersed duplicates in which one copy lacks introns; 44 in total).

### Selection Tests

We tested for positive selection using the “branch-site” test 2 implemented in codeML from PAML v.4.9 (Yang 2007). We compared the null hypothesis where d*N*/d*S* is fixed in all branches (model = 2, NSsites = 2, fix_omega = 1, and omega = 1) and the alternative hypothesis where the branch that is being tested for positive selection may include codons evolving at d*N*/d*S* > 1 (model = 2, NSsites = 2, fix_omega = 0, and omega = 1.5). The two models were compared using a likelihood ratio test, and *P* values were adjusted for multiple comparisons using the Holm, Hochberg, SidakSS, SidakSD, BH, and BY methods using multtest package for R. We considered that a given gene is under selection if any of the adjusted *P* values computed using the different methods was <0.01.

Selection tests taking into account MNMs were explored in HyPhy (Pond et al. 2020) following the pipeline described by Venkat et al. (2018). Null and alternative hypotheses were compared as described before.

We obtained population data from three different *A. pisum* host races specialized on different crops: alfalfa (population size = 60), genista (population size = 34), and pea (population size = 60) (Grigorescu et al. 2018; Nouhaud et al. 2018). To compute Tajima’s *D* for each gene, we mapped the reads with bwa and generated a pileup with samtools mpileup (Etherington et al. 2015). Polymerase chain reaction duplicates were removed and the primary alignments with a mapping quality higher than 20 were kept. From the pileup file, we run the script subsample-pileup.pl from Popoolation (Kofler et al. 2011), with the option --target-coverage 15 --max-coverage 150 --method withoutreplace --fastq-type sanger. Then, Tajima’s *D* was calculated using the script “Variance-at-position.pl --measure D” from Popoolation, with the parameter pool size set to 120, 120, and 68 for the alfalfa, pea, and genista sagittalis, respectively, on a GTF file with all the protein coding genes in the *A. pisum* genome. We computed Tajima’s *D* for whole genes, exons, and introns.

The codon adaptation index for each gene of interest was estimated using CaiCAL (Puigbò et al. 2008). The codon usage table for *A. pisum* was obtained from CoCoPUTs (Athey et al. 2017). CaiCAL was also used to calculate the expected Codon Adaptation Index (CAI) value based on a 1,000 randomly created sequences. Genes with a CAI value above the eCAI value were considered as optimized.

### Functional Annotation and GO Term Enrichment Analysis and Visualization

To assign GO terms to the genes in the pea aphid genome, GO terms based on orthology relationship were propagated with eggNOG-mapper (Huerta-Cepas et al. 2017). For that, we selected the eukaryotic eggNOG database (euNOG; Huerta-Cepas et al. 2019) and prioritized coverage (i.e., GO terms were propagated if any type of orthologs to a gene in a genome was detected). See supplementary table S9, Supplementary Material online, for the full annotation of the selected genes. Functional enrichment of the selected duplications was explored with FatiGO (Al-Shahrour et al. 2004). We tested enrichment against two different backgrounds: all the genome and the remaining genes in the genome (i.e., nonexpanded genes and nonpositively selected ones, respectively). Sets of GO terms were summarized and visualized in REVIGO (Supek et al. 2011).

### Tissue Expression Diverge between Duplicates

Messenger RNA (mRNA) expression data were obtained from 106 different samples from the *A. pisum* LSR1 lineage. They correspond to RNA-Seq libraries from 18 different conditions. Some of them were retrieved from the public databases and others newly generated for this study (supplementary table S2, Supplementary Material online). All samples were sequenced using Illumina technology as paired-end of 100-bp size, containing more than 25 million raw reads per library. Reads from all the RNA libraries were mapped on the version 2 of the pea aphid genome assembly (Acyr_2.0, ID NCBI: 246238) using STAR version 2.5.2a (Dobin et al. 2013) with the default parameters except the following parameters: outFilterMultimapNmax = 5, outFilterMismatchNmax = 3, alignIntronMin = 10, alignIntronMax = 50,000, and alignMatesGapMax = 50,000. The number of reads covering each gene prediction (NCBI annotation release ID: 102) was then counted using FeatureCounts version 1.5.0-p3 (Liao et al. 2014) with the default parameters except the following parameters: -g gene -C -p -M --fraction. For each counting, RPKM calculation was performed using edgeR (Robinson et al. 2010; McCarthy et al. 2012) with gene.length = sum of exons size for each gene. TPMs were calculated from RPKM using the equation TPM(i) = [FPKM(i)/sum (FPKM all transcripts)] × 10^6^. Principal component analysis was performed using prcomp function from R.

RNA-Seq values for each individual gene were divided into four quartiles. Each RNA-Seq experiment was processed independently. Replicates were then joined by collapsing the different values obtained in the different experiments of the same tissue. If more than 50% of the experiments placed the RNA-Seq data into the same, this bin was assigned to the overall tissue. On the other hand, if none of the bins had enough representation across experiments, no value was assigned (NA). Once each tissue was assigned a value, a profile was created for each individual gene. The profiles consisted of 0 and 1 in which 0 represented not expressed and consisted of values located in the lowest of the four bins. The value “1” represented expressed genes and consisted of values located in the other three bins. These expression profiles were used to calculate the tissue expression divergence between pairs of duplicates using three different methods: 1) Normalized Hamming distance, which counts the number of differences between two profiles and divides it by the total number of considered tissues. A tissue is not considered when its value is NA for either gene. 2) Tissue expression complementarity distance (Huerta-Cepas, Dopazo, et al. 2011), which compares the relative number of tissues in which only one set but not the other was expressed over the total number of tissues in which each gene is expressed. 3) Tissue expression divergence (d*T*) (Pegueroles et al. 2013), which subtracts tissues were one or two copies are expressed from tissues where the two copies are expressed divided by tissues where one or two copies are expressed. Values for the three distances range from 0 to 1, where 0 means no differences in gene expression between duplicates (in other words, the two copies tend to be expressed in the same tissues) and 1 means that the two copies have totally different expression patterns. In addition, for each replicate of a given tissue, we computed the median expression value in TPM. We then rescaled the expression across the tissues using the rescale function from plyr package from R and subsequently we calculated the median expression value, the Pearson correlation and its *P* value, and the *r*^2^ and the slope of a linear model using gene expression across the 18 tissues.

### FAIRE-Seq Data Analysis

FAIRE-Seq data for samples for males and females adults were taken from Richard et al. (2017). FAIRE-Seq samples for embryos were newly generated for another, unpublished, study (Richard 2017). Subsequently to sequencing, FAIRE and Control reads were mapped using bowtie2 with default parameters (Langmead et al. 2009; Langmead and Salzberg 2012) on the pea aphid genome assembly Acyr_2.0 (ID NCBI: 246238, AphidBase: http://bipaa.genouest.org/is/aphidbase/acyrthosiphon_pisum/). Only uniquely mapped reads with a mapping quality over or equal to 30 in the phred scale were kept using SAMtools (Li et al. 2009), following the irreproducible discovery rate (IDR) recommendations (Li et al. 2011, https://sites.google.com/site/anshulkundaje/projects/idr#TOC-Latest-pipeline). MACS2 (Zhang et al. 2008) was used to perform the peak calling with the following parameters using control samples: --gsize 541675471 --nomodel --extsize 500 -p 0.05 --keep-dup all -f BEDPE, followed by IDR analyses using a threshold of 0.01 for original replicates, 0.02 for self-consistency replicates, and 0.0025 for pooled pseudoreplicates. Replicates consistency was then assessed using the IDR algorithm (Li et al. 2011) and the two most correlated FAIRE replicates out of the three in each condition were pooled in order to reduce the noise, as widely recommended for ChIP-Seq or ATAC-Seq data. Input-normalized FAIRE-Seq signals were calculated using deepTools2 *bamCompare* (Ramírez et al. 2016) across the whole genome for each condition by calculating the average log 2 (Pooled FAIRE/Input) in windows of 10 bp. Both Pooled FAIRE and Input read counts were normalized by sequencing depth using “*--normalizeTo1x*.” Using deepTools2 *multiBigwigsummary*, the average FAIRE signal was extracted 900 bp around the beginning of genes (450 bp in 5′ and 450 bp in 3′) in all samples. We then used a threshold of 1 for the average log 2 (FAIRE/Input) to define genes whose TSS is open (above the threshold) or closed (below the threshold).

Embryos and adults RNA-Seq data were related to the FAIRE-Seq data for each condition and individual gene. According to the data, genes were classified in four categories: 1) open and expressed, 2) open and not expressed, 3) closed and expressed, and 4) closed and not expressed. For each gene, the percentage of tissues representing each category was calculated. If this average reached at least 75%, a single category was assigned to the gene. In total, 13,858 genes were assigned to one of the categories (supplementary table S6, Supplementary Material online).

## Supplementary Material

Supplementary data are available at *Molecular Biology and Evolution* online.

## Supplementary Material

msaa110_Supplementary_Data
