# Supplementary material for: Selection following Gene Duplication Shapes Recent Genome Evolution in the Pea Aphid Acyrthosiphon pisum
Source: Mol Biol Evol. 2020 May 2;37(9):2601–15. doi: 10.1093/molbev/msaa110 (PMC7475028; doi:10.1093/molbev/msaa110)
Supplement: msaa110_Supplementary_Data [file msaa110_supplementary_data.zip › msaa110-Suppl_Data/SuplFig16.pdf]

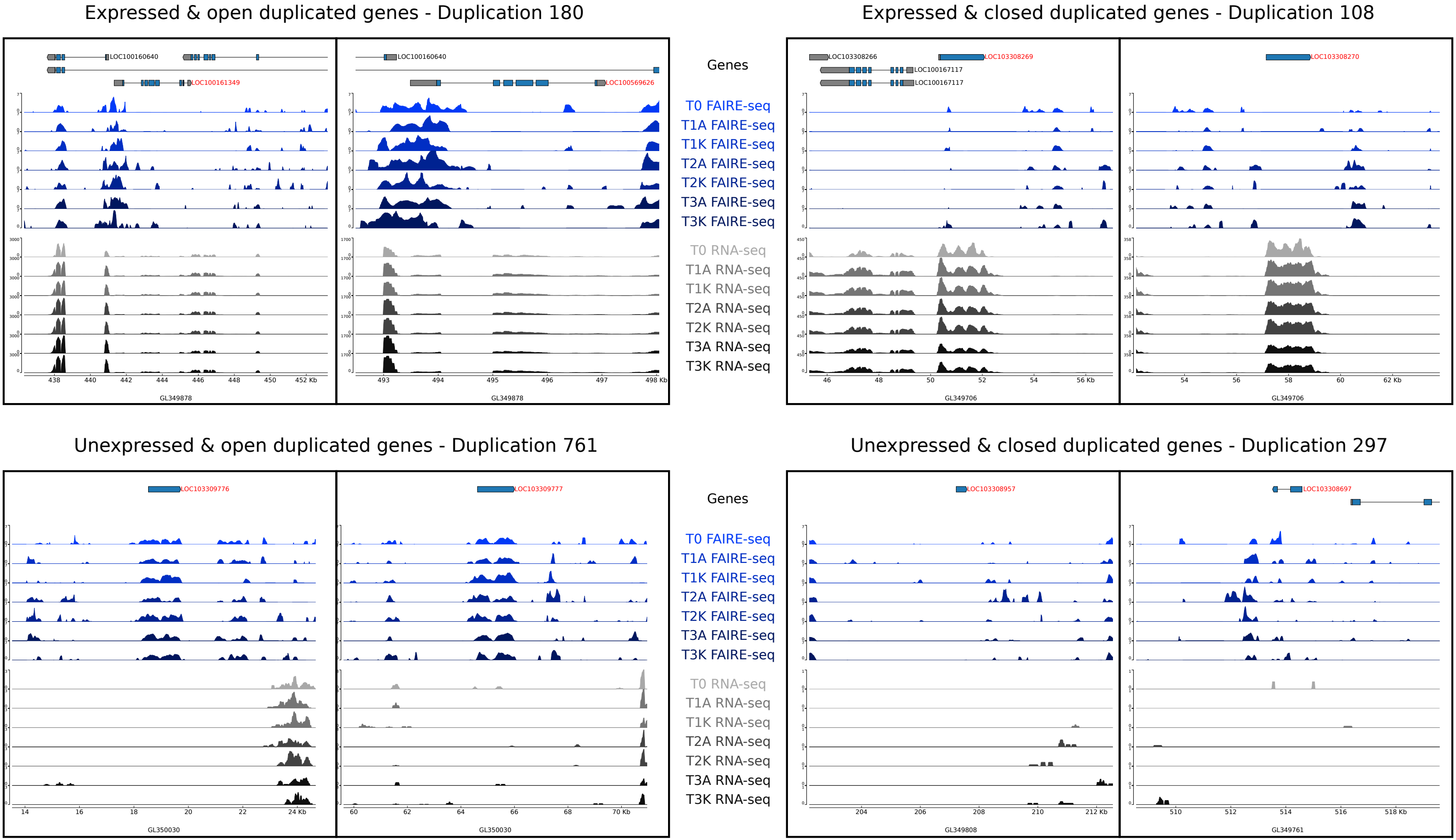

Expressed & open duplicated genes - Duplication 180
Expressed & closed duplicated genes - Duplication 108
Unexpressed & open duplicated genes - Duplication 761
Unexpressed & closed duplicated genes - Duplication 297
Genes
T0 FAIRE-seq
T1A FAIRE-seq
T1K FAIRE-seq
T2A FAIRE-seq
T2K FAIRE-seq
T3A FAIRE-seq
T3K FAIRE-seq
T0 RNA-seq
T1A RNA-seq
T1K RNA-seq
T2A RNA-seq
T2K RNA-seq
T3A RNA-seq
T3K RNA-seq
LOC100160640
LOC100161349
LOC100569626
LOC103308266
LOC103308269
LOC100167117
LOC103308270
LOC103309776
LOC103309777
LOC103308957
LOC103308697
GL349878
GL349706
GL350030
GL349808
GL349761
