# Supplementary figures and images for: Selection following Gene Duplication Shapes Recent Genome Evolution in the Pea Aphid Acyrthosiphon pisum
Source: Mol Biol Evol. 2020 May 2;37(9):2601–15. doi: 10.1093/molbev/msaa110 (PMC7475028; doi:10.1093/molbev/msaa110)

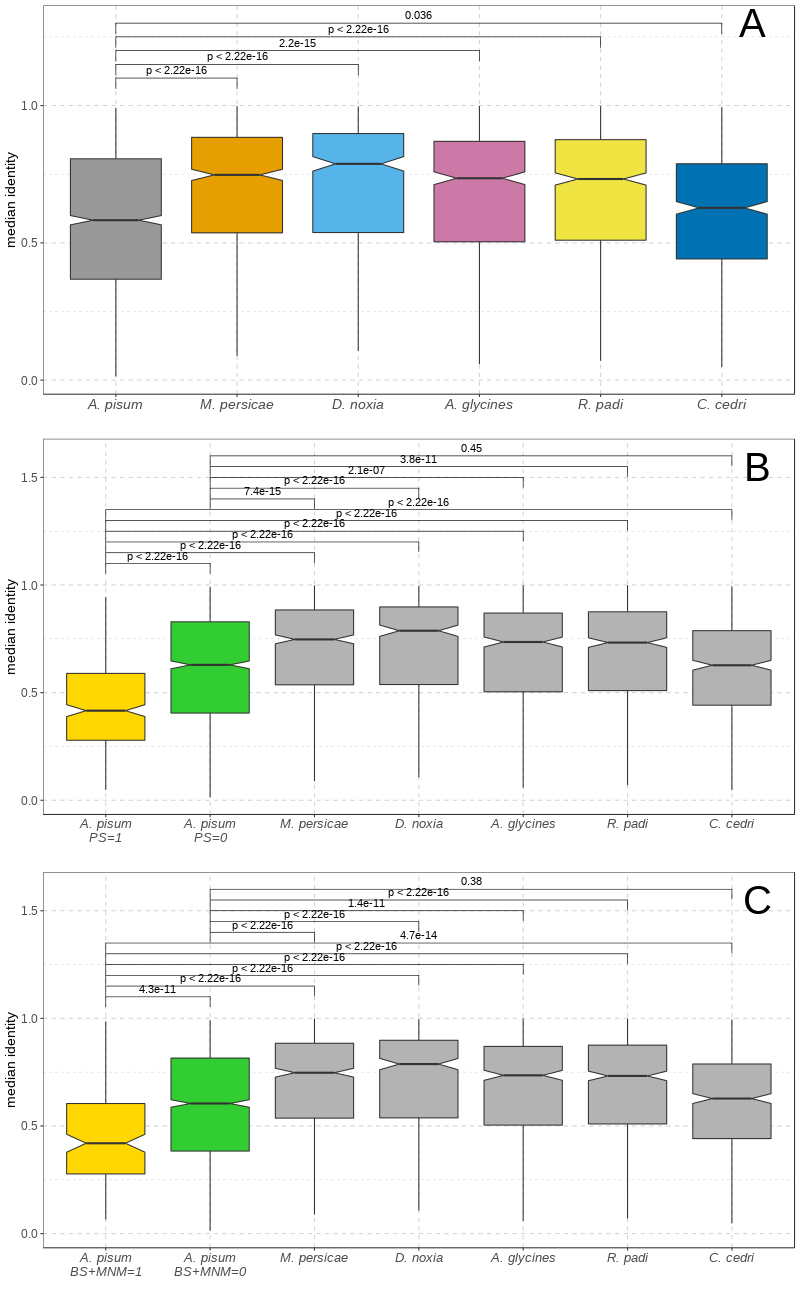

Supplement: msaa110_Supplementary_Data [file msaa110_supplementary_data.zip › msaa110-Suppl_Data/SuplFig1.jpg]

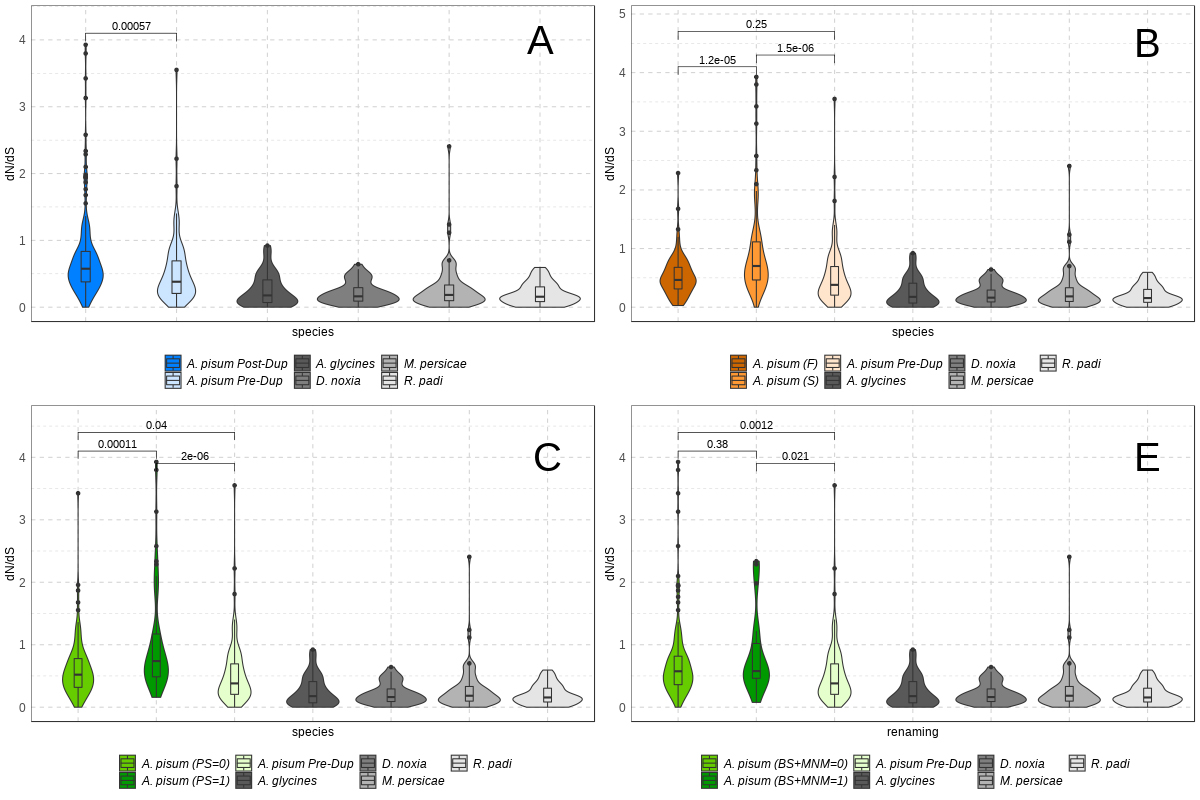

Supplement: msaa110_Supplementary_Data [file msaa110_supplementary_data.zip › msaa110-Suppl_Data/SuplFig2.jpg]

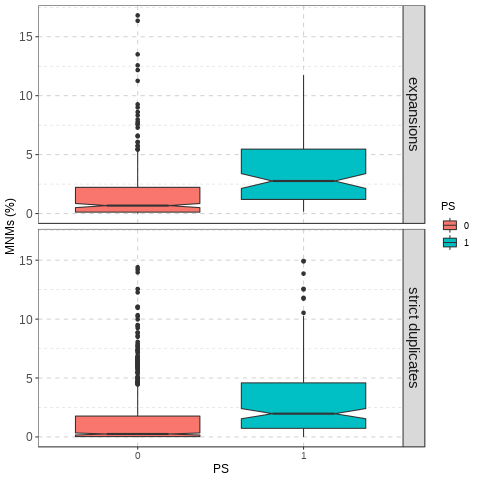

Supplement: msaa110_Supplementary_Data [file msaa110_supplementary_data.zip › msaa110-Suppl_Data/SuplFig3.jpg]

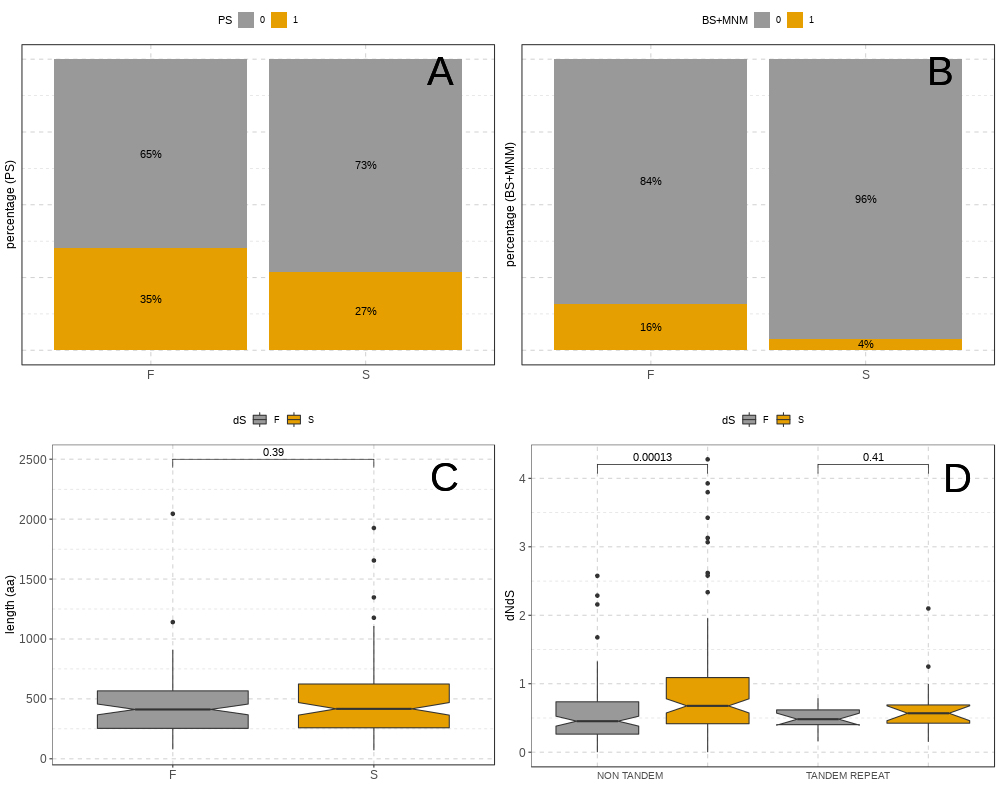

Supplement: msaa110_Supplementary_Data [file msaa110_supplementary_data.zip › msaa110-Suppl_Data/SuplFig4.jpg]

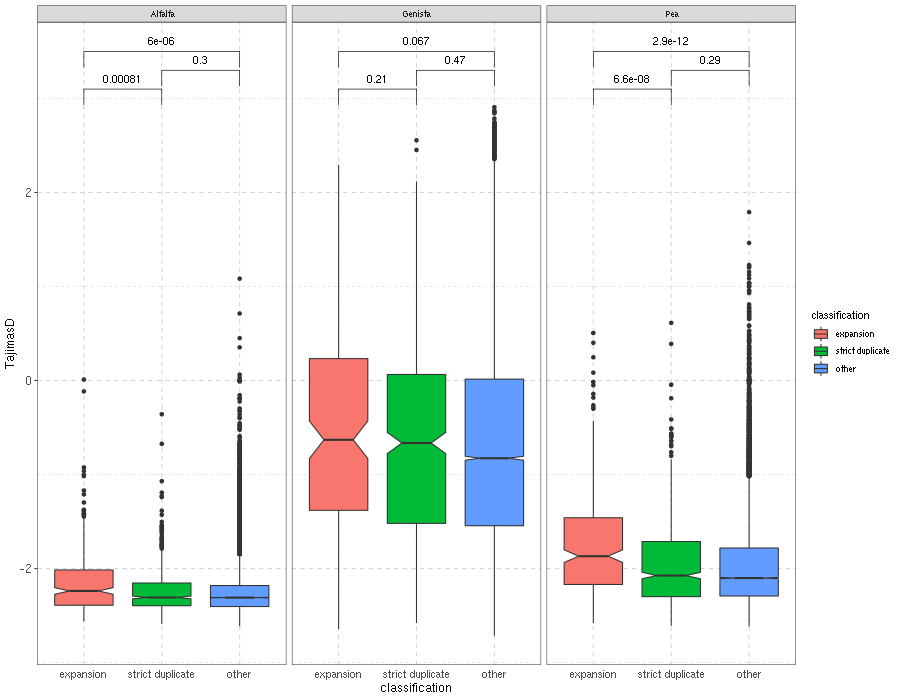

Supplement: msaa110_Supplementary_Data [file msaa110_supplementary_data.zip › msaa110-Suppl_Data/SuplFig5.jpg]

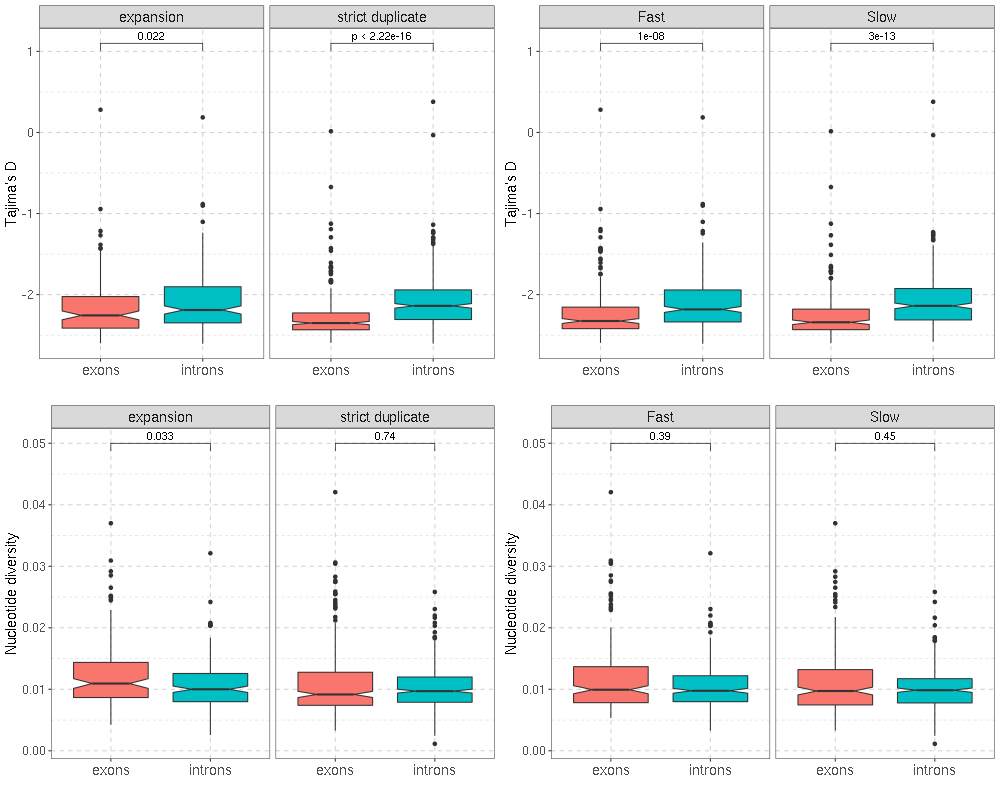

Supplement: msaa110_Supplementary_Data [file msaa110_supplementary_data.zip › msaa110-Suppl_Data/SuplFig6.jpg]

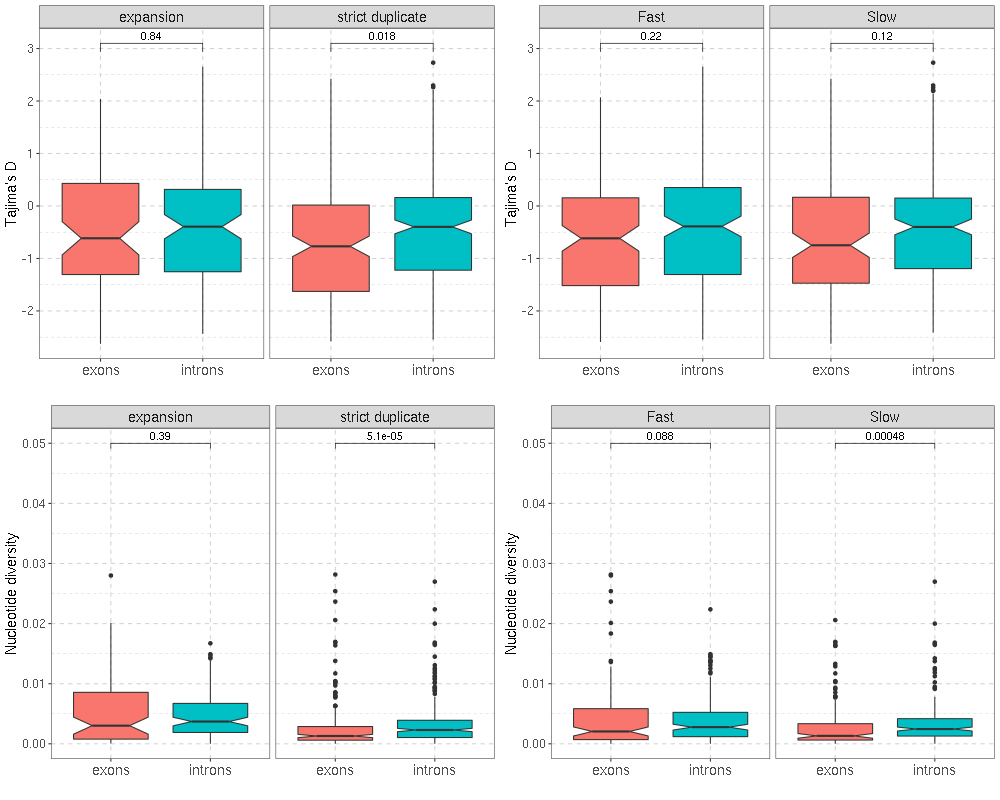

Supplement: msaa110_Supplementary_Data [file msaa110_supplementary_data.zip › msaa110-Suppl_Data/SuplFig7.jpg]

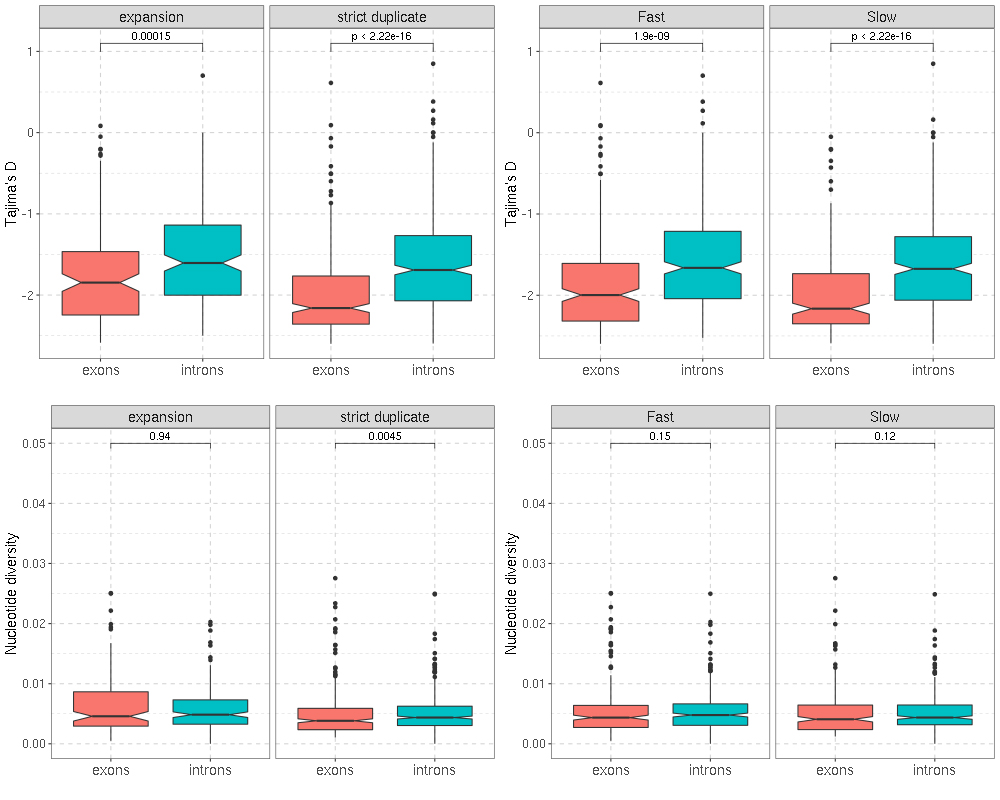

Supplement: msaa110_Supplementary_Data [file msaa110_supplementary_data.zip › msaa110-Suppl_Data/SuplFig8.jpg]

REVIGO Gene Ontology treemap

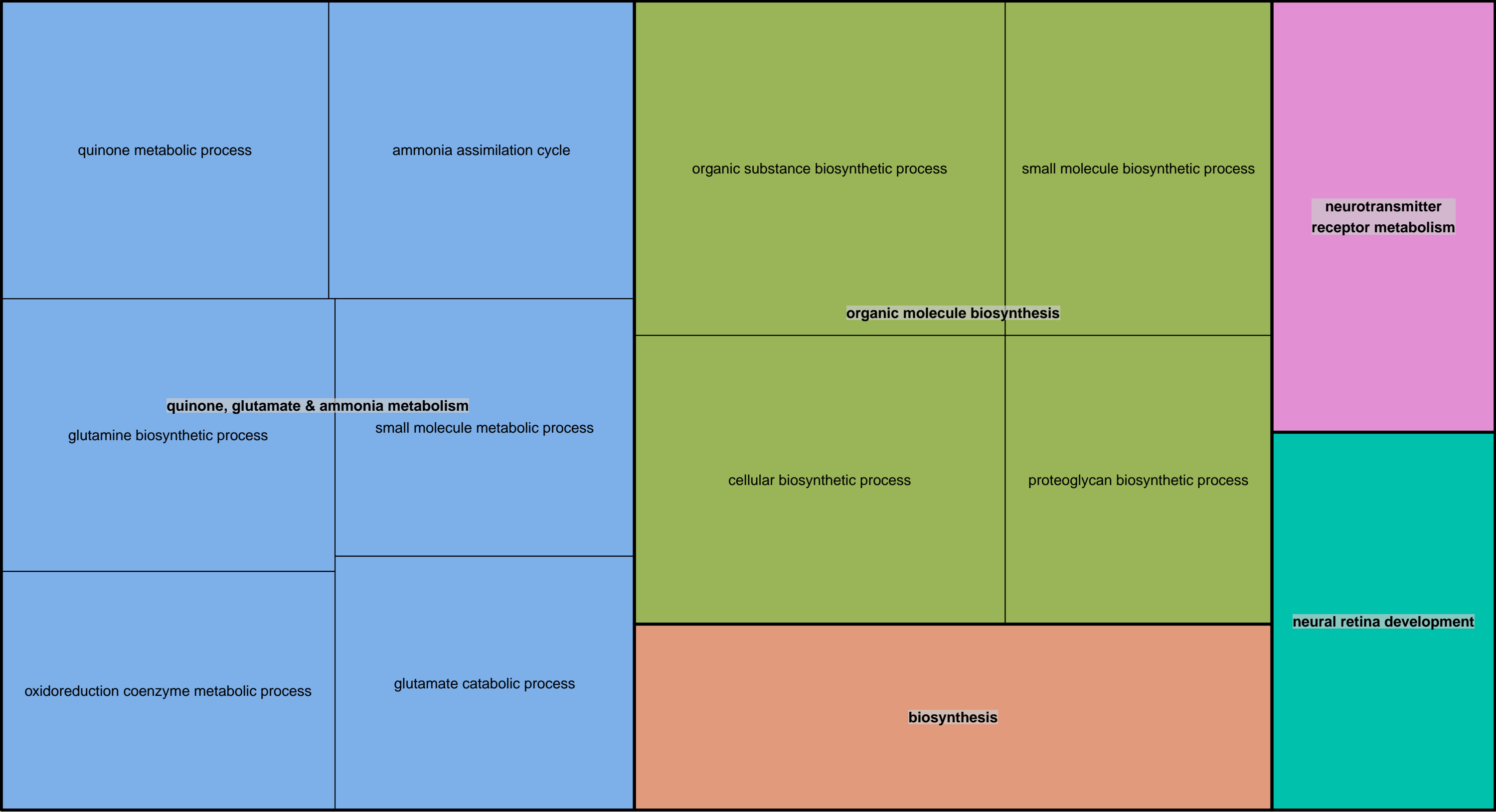

Supplement: msaa110_Supplementary_Data [file msaa110_supplementary_data.zip › msaa110-Suppl_Data/SuplFig9.pdf]

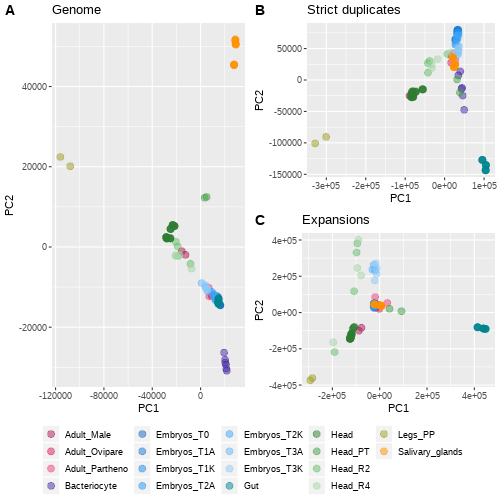

Supplement: msaa110_Supplementary_Data [file msaa110_supplementary_data.zip › msaa110-Suppl_Data/SuplFig10.jpg]

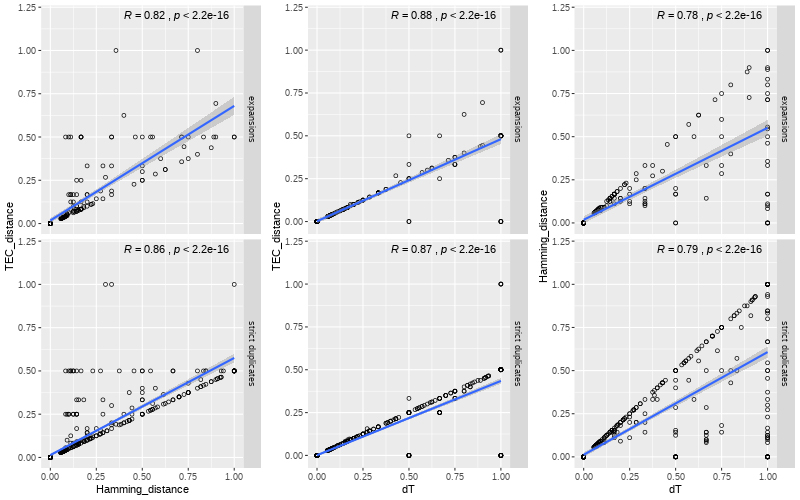

Supplement: msaa110_Supplementary_Data [file msaa110_supplementary_data.zip › msaa110-Suppl_Data/SuplFig11.jpg]

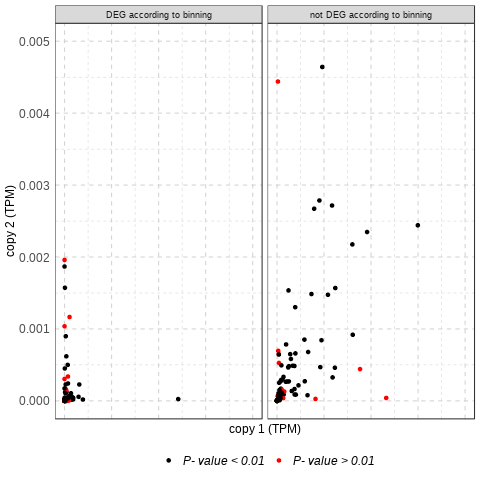

Supplement: msaa110_Supplementary_Data [file msaa110_supplementary_data.zip › msaa110-Suppl_Data/SuplFig12.jpg]

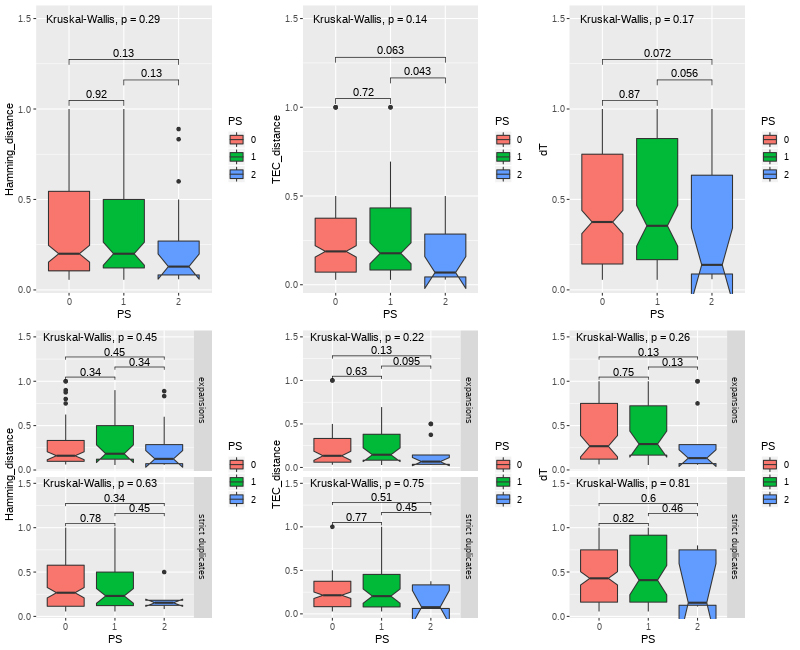

Supplement: msaa110_Supplementary_Data [file msaa110_supplementary_data.zip › msaa110-Suppl_Data/Suplfig13.jpg]

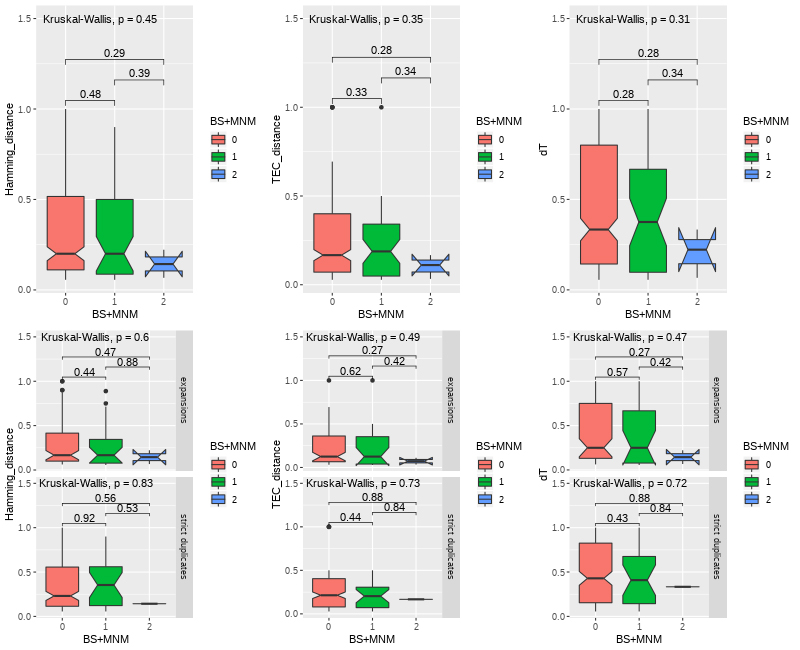

Supplement: msaa110_Supplementary_Data [file msaa110_supplementary_data.zip › msaa110-Suppl_Data/SuplFig14.jpg]

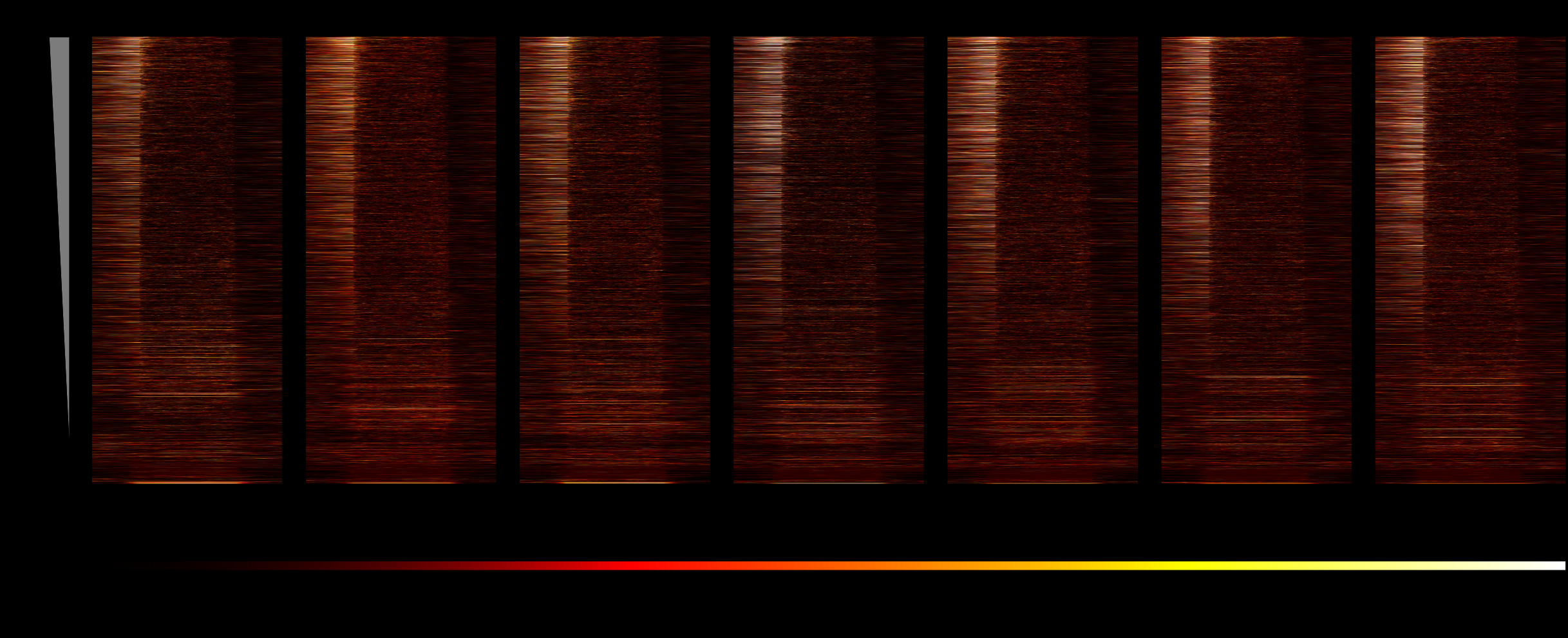

Supplement: msaa110_Supplementary_Data [file msaa110_supplementary_data.zip › msaa110-Suppl_Data/SuplFig15.jpg]
